# Non-Iterative Multiscale Estimation for Spatial Autoregressive Geographically Weighted Regression Models

**DOI:** 10.3390/e25020320

**Published:** 2023-02-09

**Authors:** Shi-Jie Gao, Chang-Lin Mei, Qiu-Xia Xu, Zhi Zhang

**Affiliations:** 1 Department of Finance and Statistics, School of Science, Xi’an Polytechnic University, Xi’an 710048, China; 2 Department of Statistics, School of Mathematics and Statistics, Xi’an Jiaotong University, Xi’an 710049, China

**Keywords:** geographically weighted regression, spatial autoregressive geographically weighted regression model, multiscale estimation, spatial scale

## Abstract

Multiscale estimation for geographically weighted regression (GWR) and the related models has attracted much attention due to their superiority. This kind of estimation method will not only improve the accuracy of the coefficient estimators but also reveal the underlying spatial scale of each explanatory variable. However, most of the existing multiscale estimation approaches are backfitting-based iterative procedures that are very time-consuming. To alleviate the computation complexity, we propose in this paper a non-iterative multiscale estimation method and its simplified scenario for spatial autoregressive geographically weighted regression (SARGWR) models, a kind of important GWR-related model that simultaneously takes into account spatial autocorrelation in the response variable and spatial heterogeneity in the regression relationship. In the proposed multiscale estimation methods, the two-stage least-squares (2SLS) based GWR and the local-linear GWR estimators of the regression coefficients with a shrunk bandwidth size are respectively taken to be the initial estimators to obtain the final multiscale estimators of the coefficients without iteration. A simulation study is conducted to assess the performance of the proposed multiscale estimation methods, and the results show that the proposed methods are much more efficient than the backfitting-based estimation procedure. In addition, the proposed methods can also yield accurate coefficient estimators and such variable-specific optimal bandwidth sizes that correctly reflect the underlying spatial scales of the explanatory variables. A real-life example is further provided to demonstrate the applicability of the proposed multiscale estimation methods.

## 1. Introduction

Geographically weighted regression (GWR) models [1,2,3], which are an extension of the linear regression models by allowing the regression coefficients to vary over space, have been a popular tool for modeling spatial heterogeneity in regression relationships. A GWR model is originally calibrated by the locally weighted least-squares procedure, where the local weights at each focal spatial location are determined by a pre-specified kernel function with a single bandwidth for all of the regression coefficients, and the optimal bandwidth size is chosen by a data-driven criterion, such as the cross-validation (CV) or the corrected Akaike information criterion (AICc) [2,3]. In the GWR technique, spatial heterogeneity in regression relationships is revealed by spatial variation patterns of the estimated regression coefficients. Therefore, the accuracy of coefficient estimators is essential to validly interpret spatial heterogeneity in regression relationships.

In geographical information science, spatial scale is one of the most important concepts [4], and a spatial process inherently operates at a spatial scale [5]. In a GWR model, different spatial scales of the explanatory variables may lead to the fact that their respective coefficients have different levels of spatial heterogeneity, and a common bandwidth in the traditional GWR technique can not produce valid estimators for all of the coefficients [6,7], which has also been theoretically proven in statistically varying coefficient models [8]. In order to overcome this shortcoming, Yang [6] proposed GWR with flexible bandwidths in which the backfitting procedure [9] is employed to iteratively estimate the spatially varying coefficients with the variable-specific bandwidth sizes selected by the AICc or CV criterion. Furthermore, Fotheringham et al. [7] explicitly connected the spatial scale of each explanatory variable with its specific bandwidth and termed the model as a multiscale geographically weighted regression model. A similar calibration procedure was also suggested by Leong and Yue [10] from the perspective of improving the coefficient estimation accuracy of a GWR model. For ease of presentation, we henceforth refer to this multiscale estimation method as GWR-BF, meaning that a GWR model is calibrated by the backfitting-based iterative procedure. It has been demonstrated that GWR-BF will yield not only more accurate estimators of the regression coefficients but also provide information about the spatial scale at which each explanatory variable operates [7,10,11].

Recently, GWR-BF has been extended to some other GWR-related models. For example, Chen and Mei [12] extended the GWR-BF method and formulated a multiscale estimation procedure for the semi-parametric GWR models originally proposed by Brunsdon et al. [13]; furthermore, combining the backfitting procedure with the profile maximum likelihood estimation for spatial autoregressive geographically weighted regression (SARGWR) models [14,15,16], Chen et al. [17] formulated a multiscale estimation method for the models; Wu et al. [18] proposed a backfitting-based multiscale estimation approach for geographically and temporally weighted regression (GTWR) models [19,20]; Zhang et al. [21] suggested a unilateral temporal weighting scheme and proposed a more flexible multiscale estimation method for GTWR models. Due to its superiority, the multiscale estimation for the GWR and the related models have been applied to many areas for spatial or spatiotemporal data analysis (see, for example, [22,23,24,25,26,27,28]).

The backfitting-based multiscale estimation methods for the GWR and the related models, however, are iterative algorithms in estimating individual regression coefficients and searching for their respective optimal bandwidth sizes. Therefore, such methods are very time-consuming, especially when the number of explanatory variables is large. In particular, the multiscale estimation method for SARGWR models in Chen et al. [17] is much more time-demanding because, in addition to the computation cost of the iterative backfitting algorithm and maximum likelihood estimation, the spatial autoregressive coefficient should also be optimized in each iteration loop, where the determinant of the related matrix with its order being the sample size needs to be calculated for each candidate value of the autoregressive coefficient, although this maximum-likelihood-based multiscale estimation can yield more accurate estimators for both the autoregressive and the regression coefficients. Therefore, the development of cost-effective algorithms is essential for real-world applications of multiscale estimation methods of the GWR and its related models.

Recently, there has been a rapid development of machine-learning-based GWR methods, such as geographically weighted extreme learning machine [29], geographically weighted elastic net [30], geographically (and temporally) neural network weighted regression [31,32], geographically weighted regression with the integration of machine learning [33], and the adapted geographically LASSO [34]. One can refer to the reference [35] for a comprehensive overview of spatial machine learning methods, including GWR models. The machine-learning-based GWR methods are, in general, more efficient in estimation and accurate in prediction than the multiscale and even the traditional calibration methods. Nevertheless, how to derive the spatial scale information of each explanatory variable with machine-learning-based GWR methods remains to be investigated.

Focusing on the GWR-BF multiscale estimation procedure, some efforts have been devoted to the reduction of computation cost. For example, Yang [6] suggested to pre-specify a larger value of the convergence threshold. However, this may lead to the premature of coefficient estimators. Based on the power of modern computers, Li and Fotheringham [36] formulated a parallel implementation for GWR-BF. Very recently, Wu et al. [37] proposed a non-iterative multiscale estimation procedure for GWR models. In this estimation method, the idea of the two-step locally weighted least-squares procedure proposed by Fan and Zhang [8] for fitting the statistically varying coefficient models is employed to implement the non-iterative estimation and the local-linear-fitting method for calibrating GWR models [38] is further used in each step to reduce the boundary effect of coefficient estimators. We henceforth abbreviate this non-iterative multiscale estimation method as GWR-LL, where “LL” means the coefficients are estimated by the local-linear-fitting method in both steps. The simulation study with a real-life example in Wu et al. [37] shows that GWR-LL is not only much more cost-effective than GWR-BF but can also significantly reduce the boundary effect of coefficient estimators. Furthermore, GWR-LL also yields such variable-specific optimal bandwidth sizes that correctly characterize the respective spatial scales of the explanatory variables.

As a kind of important GWR-related model, the spatial autoregressive geographically weighted regression (SARGWR) model incorporates a spatial lag term of the response variable into the GWR model to simultaneously take into account spatial autocorrelation in the response variable and spatial heterogeneity in the regression relationships [14,15,16]. Since SARGWR models can simultaneously consider the two fundamental properties of spatial data, i.e., spatial autocorrelation and spatial heterogeneity, they have a wide application background in spatial data analysis. Therefore, it is of great importance to the development of some cost-effective multiscale estimation methods in view of the advantages of the multiscale estimation methods for calibrating the GWR and its related models. As aforementioned, however, the existing iterative multiscale estimation for SARGWR models proposed by Chen et al. [17] is extremely time-consuming.

Motivated by the simplicity of the two-stage least-squares (2SLS) method in which the spatial lag term is replaced by an instrument-variables-based estimator, and then the autoregressive coefficient is estimated by the ordinary least-squares procedure [39,40], and considering that the GWR-based 2SLS estimation for SARGWR models has been explicitly formulated in Mei and Chen [41], we first extend in this paper the GWR-based 2SLS estimation to the local-linear GWR-based 2SLS estimation of SARGWR models. Then, the idea of GWR-LL in Wu et al. [37] is employed to develop a non-iterative multiscale estimation method for SARGWR models. Specifically, the extended local-linear GWR-based 2SLS estimation, instead of the profile maximum likelihood estimation in Chen et al. [17], of SARGWR models are used to derive the initial estimators of the spatial autoregressive coefficient and the regression coefficients for the purpose of reducing the computation cost. Then, the GWR-LL procedure is applied to the estimation of the regression coefficients. Furthermore, the 2SLS estimation for spatial autoregressive models [39,40] is used to re-estimate the autoregressive coefficient. This non-iterative multiscale estimation method is referred to as SARGWR-LL henceforth, meaning that the local-linear GWR estimation method [38] is used in both the initial and final steps. Moreover, considering that the local-linear estimation of GWR models is more time-consuming than their traditional estimation, and the initial estimators of the regression coefficients might have less influence on their final multiscale estimators, we further propose a simplified scenario of SARGWR-LL by replacing the initial local-linear GWR-based 2SLS estimators of the spatial autoregressive coefficient and regression coefficients with their respective GWR-based 2SLS estimators, which we refer to as SARGWR-GL in the subsequent presentation, where “G” means that the GWR-based 2SLS estimation method is employed in the initial step.

The rest of the paper is organized as follows. In Section 2, the GWR-based 2SLS estimation of SARGWR models [41] is briefly reviewed, on which its local-linear GWR-based version is derived; based on the GWR-based 2SLS estimation and its local-linear GWR-based version of SARGWR models, the SARGWR-LL and its simplified scenario SARGWR-GL are finally formulated. A simulation study and a real-life example based on Dublin voter turnout data are conducted in Section 3 to assess and compare the performance of the related multiscale estimation methods for SARGWR models. The paper is ended with a conclusion and discussion.

## 2. Methods

### 2.1. Spatial Autoregressive Geographically Weighted Regression (SARGWR) Model

Let *Y* be the response variable, and $X_{1},X_{2},\cdots ,X_{p}$ be the associated explanatory variables. Given their observations ${\left\{\left(y_{i};x_{i1},x_{i2},\cdots ,x_{ip}\right)\right\}}_{i=1}^{n}$ collected at *n* spatial locations ${\left\{\left(u_{i},v_{i}\right)\right\}}_{i=1}^{n}$, the SARGWR model studied in this paper is

(1) $$y_{i}=\rho \sum_{j=1}^{n}w_{ij}y_{j}+\sum_{j=1}^{p}\beta_{j}\left(u_{i},v_{i}\right)x_{ij}+\varepsilon_{i},\;i=1,2,\cdots ,n,$$

where the parameter ρ, which is assumed to be |ρ|<1, is called the autoregressive coefficient, measuring the intensity of spatial autocorrelation in the response variable *Y*; ${\left\{w_{ij}\right\}}_{i,j=1}^{n}$ are the elements of a pre-specified row-standardized spatial weights matrix $W={\left(w_{ij}\right)}_{n\times n}$ of the *n* sampling locations with $w_{ii}=0\;\left(i=1,2,\cdots ,n\right)$ assumed by convention, which characterizes the neighborhood relationship between each sampling point and its neighbors; ${\left\{\beta_{j}\left(u,v\right)\right\}}_{j=1}^{p}$ are *p* regression coefficients which are unknown functions of the spatial coordinates (u,v); and ${\left\{\varepsilon_{i}\right\}}_{i=1}^{n}$ are independent and identically distributed errors with $E(\varepsilon_{i})=0$ and $\mathrm{Var}\left(\varepsilon_{i}\right)=\sigma^{2}>0$. We can take $X_{1}=1$ (i.e., $x_{i1}=1$ for i=1,2,⋯,n) to make the model include a spatially varying intercept.

Let $y={\left(y_{1},y_{2},\cdots ,y_{n}\right)}^{T}$; $x_{i}={\left(x_{i1},x_{i2},\cdots ,x_{ip}\right)}^{T}\left(i=1,2,\cdots ,n\right)$; $\varepsilon ={\left(\varepsilon_{1},\varepsilon_{2},\cdots ,\varepsilon_{n}\right)}^{T}$; $\beta \left(u_{i},v_{i}\right)={\left(\beta_{1}\left(u_{i},v_{i}\right),\beta_{2}\left(u_{i},v_{i}\right),\cdots ,\beta_{p}\left(u_{i},v_{i}\right)\right)}^{T}\left(i=1,2,\cdots ,n\right)$; and $M=\left(x_{1}^{T}\beta \left(u_{1},v_{1}\right),\right.{\left.x_{2}^{T}\beta \left(u_{2},v_{2}\right),\cdots ,x_{n}^{T}\beta \left(u_{n},v_{n}\right)\right)}^{T}$. The SARGWR model in Equation (1) can be expressed by matrix notation as

(2) y=ρWy+M+ε.

**Remark 1**. *The above SARGWR model assumes that the autoregressive coefficient ρ is constant. This means that spatial autocorrelation in the response variable between the observation of the response variable at any spatial sampling unit and those observed at its neighbors is the same over space, which may be unrealistic for many real-life spatial data sets. The more general SARGWR model should allow ρ to vary over space, i.e., ρ(u,v) instead. Considering that the SARGWR model with constant autoregressive coefficient is a kind of standard model in the application, and the 2SLS procedure can be directly employed to estimate the parameter ρ, we then mainly focus this kind of SARGWR model on deriving its non-iterative multiscale estimation method. The extension to the SARGWR model with a spatially varying autoregressive coefficient ρ(u,v) will be discussed in the final section.*

### 2.2. Preliminary Methods for Formulating the Non-Iterative Multiscale Estimation of the SARGWR Model

#### 2.2.1. GWR-Based 2SLS Estimation of the SARGWR Model

In this subsection, we briefly review the GWR-based 2SLS estimation of the SARGWR model, which we will use to formulate the SARGWR-GL multiscale estimation procedure. For more detailed derivation, one can refer to the supplementary materials at https://doi.org/10.1016/j.spasta.2022.100666 provided in Mei and Chen [41].

We rewrite the model in Equation (2) as

(3) $$\tilde{y}\overset{\wedge }{=}y-\rho \mathrm{Wy}=M+\varepsilon ={\left(x_{1}^{T}\beta \left(u_{1},v_{1}\right),x_{2}^{T}\beta \left(u_{2},v_{2}\right),\cdots ,x_{n}^{T}\beta \left(u_{n},v_{n}\right)\right)}^{T}+\varepsilon .$$

We treat the above model as a GWR model with the observation vector of the response variable being $\tilde{y}=y-\rho \mathrm{Wy}$, and obtain, according to Brunsdon et al. [2], that the GWR estimators of $\beta \left(u_{i},v_{i}\right)\;\left(i=1,2,\cdots ,n\right)$ are

(4) $$\begin{array}{cc} {\hat{\beta }}_{G}\left(u_{i},v_{i}\right) & ={\left({\hat{\beta }}_{G1}\left(u_{i},v_{i}\right),{\hat{\beta }}_{G2}\left(u_{i},v_{i}\right),\cdots ,{\hat{\beta }}_{Gp}\left(u_{i},v_{i}\right)\right)}^{T}\\ \; & ={\left(X^{T}W_{h}\left(u_{i},v_{i}\right)X\right)}^{-1}X^{T}W_{h}\left(u_{i},v_{i}\right)\left(y-\rho \mathrm{Wy}\right),\;i=1,2,\cdots ,n, \end{array}$$

where the subscript “G” means the traditional GWR estimation method, $X={\left(x_{1},x_{2},\cdots ,x_{n}\right)}^{T}$ is the design matrix, and

(5) $$W_{h}\left(u_{i},v_{i}\right)=\mathrm{Diag}\left(w_{1h}\left(u_{i},v_{i}\right),w_{2h}\left(u_{i},v_{i}\right),\cdots ,w_{nh}\left(u_{i},v_{i}\right)\right)$$

is the calibration weights matrix at $\left(u_{i},v_{i}\right)$, which is related to the bandwidth *h*. Here, the word “calibration” is used in order to distinguish this matrix from the spatial weights matrix W in the model.

Substituting ${\hat{\beta }}_{G}\left(u_{i},v_{i}\right)\;\left(i=1,2,\cdots ,n\right)$ into M, we obtain the estimator of M as

$$\begin{array}{c} {\hat{M}}_{G}={\left(x_{1}^{T}{\hat{\beta }}_{G}\left(u_{1},v_{1}\right),x_{2}^{T}{\hat{\beta }}_{G}\left(u_{2},v_{2}\right),\cdots ,x_{n}^{T}{\hat{\beta }}_{G}\left(u_{n},v_{n}\right)\right)}^{T}=S_{G}\left(h\right)\tilde{y}=S_{G}\left(h\right)\left(I_{n}-\rho W\right)y, \end{array}$$

where $I_{n}$ is the identity matrix of order *n*, and

$$\begin{array}{c} S_{G}\left(h\right)=\left(\begin{array}{c} x_{1}^{T}{\left(X^{T}W_{h}\left(u_{1},v_{1}\right)X\right)}^{-1}X^{T}W_{h}\left(u_{1},v_{1}\right)\\ x_{2}^{T}{\left(X^{T}W_{h}\left(u_{2},v_{2}\right)X\right)}^{-1}X^{T}W_{h}\left(u_{2},v_{2}\right)\\ \vdots \\ x_{{}^{n}}^{T}{\left(X^{T}W_{h}\left(u_{n},v_{n}\right)X\right)}^{-1}X^{T}W_{h}\left(u_{n},v_{n}\right) \end{array}\right). \end{array}$$

Furthermore, replacing M in Equation (3) with its estimator ${\hat{M}}_{G}$ yields the following artificial spatial autoregressive model:

(6) $$\left(I_{n}-S_{G}\left(h\right)\right)y=\rho \left(I_{n}-S_{G}\left(h\right)\right)\mathrm{Wy}+\varepsilon .$$

In the above model, since Wy is endogenous, an instrumental estimator of Wy is needed to derive a consistent estimator of ρ in the framework of least-squares estimation. As suggested by Geniaux and Martinetti [16] as well as by Mei and Chen [41], the instrumental variables $Q=(X,WX_{\left(-1\right)},W^{2}X_{\left(-1\right)})$, where X is the design matrix, and $X_{\left(-1\right)}$ is such a matrix that the first column of X is removed when an intercept is included in the SARGWR model, can be used to estimate Wy by formulating the following GWR model:

(7) $$y_{wi}\overset{\wedge }{=}\sum_{j=1}^{n}w_{ij}y_{j}=q_{i}^{T}\alpha \left(u_{i},v_{i}\right)+\eta_{i},\;i=1,2,\cdots ,n,$$

where $y_{wi}$ is the *i*-th element of Wy and $q_{i}^{T}=\left(1,q_{i2},\cdots ,q_{i,3p-2}\right)$ is the *i*-th row of the instrumental variables Q. Calibrating the model in Equation (7) with the traditional GWR technique [2], we obtain the fitted values of $y_{wi}\;\left(i=1,2,\cdots ,n\right)$ as

$$\begin{array}{c} {\hat{y}}_{wi}=q_{i}^{T}{\left(Q^{T}W_{h}\left(u_{i},v_{i}\right)Q\right)}^{-1}Q^{T}W_{h}\left(u_{i},v_{i}\right)\mathrm{Wy},\;i=1,2,\cdots ,n. \end{array}$$

Therefore, the instrumental estimator of Wy, which we denoted by ${\hat{y}}_{w}$, is

(8) $${\hat{y}}_{w}={\left({\hat{y}}_{w1},{\hat{y}}_{w2},\cdots ,{\hat{y}}_{wn}\right)}^{T}=S_{Q}\left(h\right)\mathrm{Wy},$$

where

(9) $$S_{Q}\left(h\right)=\left(\begin{array}{c} q_{1}^{T}{\left(Q^{T}W_{h}\left(u_{1},v_{1}\right)Q\right)}^{-1}Q^{T}W_{h}\left(u_{1},v_{1}\right)\\ q_{2}^{T}{\left(Q^{T}W_{h}\left(u_{2},v_{2}\right)Q\right)}^{-1}Q^{T}W_{h}\left(u_{2},v_{2}\right)\\ \vdots \\ q_{n}^{T}{\left(Q^{T}W_{h}\left(u_{n},v_{n}\right)Q\right)}^{-1}Q^{T}W_{h}\left(u_{n},v_{n}\right) \end{array}\right).$$

Replacing Wy in the model in Equation (6) with its instrumental estimator ${\hat{y}}_{w}$ in Equation (8) and using the least-squares estimation procedure, we obtain the GWR-based 2SLS estimator of the autoregressive coefficient ρ as

(10) $${\hat{\rho }}_{G}={\left({\hat{y}}_{w}^{T}{\left(I_{n}-S_{G}\left(h\right)\right)}^{T}\left(I_{n}-S_{G}\left(h\right)\right){\hat{y}}_{w}\right)}^{-1}{\hat{y}}_{w}^{T}{\left(I_{n}-S_{G}\left(h\right)\right)}^{T}\left(I_{n}-S_{G}\left(h\right)\right)y.$$

Substituting ${\hat{\rho }}_{G}$ into Equation (4), we obtain the GWR-based 2SLS estimators of the regression coefficient vectors $\beta \left(u_{i},v_{i}\right)\;\left(i=1,2,\cdots ,n\right)$ as

(11) $$\begin{array}{cc} {\hat{\beta }}_{G}\left(u_{i},v_{i}\right) & ={\left({\hat{\beta }}_{G1}\left(u_{i},v_{i}\right),{\hat{\beta }}_{G2}\left(u_{i},v_{i}\right),\cdots ,{\hat{\beta }}_{Gp}\left(u_{i},v_{i}\right)\right)}^{T}\\ \; & ={\left(X^{T}W_{h}\left(u_{i},v_{i}\right)X\right)}^{-1}X^{T}W_{h}\left(u_{i},v_{i}\right)\left(I_{n}-{\hat{\rho }}_{G}W\right)y,\;i=1,2,\cdots ,n. \end{array}$$

Furthermore, the fitted values of the response variable *Y* at ${\left\{\left(u_{i},v_{i}\right)\right\}}_{i=1}^{n}$ are

$$\begin{array}{c} {\hat{y}}_{Gi}\left(h\right)={\hat{\rho }}_{G}\sum_{j=1}^{n}w_{ij}y_{j}+\sum_{j=1}^{p}{\hat{\beta }}_{Gj}\left(u_{i},v_{i}\right)\;x_{ij},\;i=1,2,\cdots ,n, \end{array}$$

and the fitted vector can be expressed as

(12) $${\hat{y}}_{G}\left(h\right)={\left({\hat{y}}_{G1}\left(h\right),{\hat{y}}_{G2}\left(h\right),\cdots ,{\hat{y}}_{Gn}\left(h\right)\right)}^{T}=H_{G}\left(h\right)y,$$

where the hat matrix is

(13) $$\begin{array}{cc} H_{G}\left(h\right)= & \left(I_{n}-S_{G}\left(h\right)\right)\left(\mathrm{Wy}\right){\left({\hat{y}}_{w}^{T}{\left(I_{n}-S_{G}\left(h\right)\right)}^{T}\left(I_{n}-S_{G}\left(h\right)\right){\hat{y}}_{w}\right)}^{-1}\\ \; & \times {\hat{y}}_{w}^{T}{\left(I_{n}-S_{G}\left(h\right)\right)}^{T}\left(I_{n}-S_{G}\left(h\right)\right)+S_{G}\left(h\right). \end{array}$$

For ease of presentation, we henceforth refer to the GWR-based 2SLS estimators ${\hat{\rho }}_{G}$ and ${\hat{\beta }}_{G}\left(u_{i},v_{i}\right)\;\left(i=1,2,\cdots ,n\right)$ in Equations (10) and (11) as their respective GWR-2SLS estimators.

#### 2.2.2. Local-Linear GWR-Based 2SLS Estimation of the SARGWR Model

In what follows, we extend the GWR-based 2SLS estimation of the SARGWR model to local-linear GWR-based estimation, which will be used to formulate the multiscale estimation procedure, SARGWR-LL.

Treating once again the model in Equation (3) as a GWR model with the observation vector of the response variable being $\tilde{y}=y-\rho \mathrm{Wy}$. According to Wang et al. [38], the local-linear estimators of $\beta \left(u_{i},v_{i}\right)\;\left(i=1,2,\cdots ,n\right)$ are

(14) $$\begin{array}{cc} {\hat{\beta }}_{L}\left(u_{i},v_{i}\right)= & {\left({\hat{\beta }}_{L1}\left(u_{i},v_{i}\right),{\hat{\beta }}_{L2}\left(u_{i},v_{i}\right),\cdots ,{\hat{\beta }}_{Lp}\left(u_{i},v_{i}\right)\right)}^{T}\\ = & \left(I_{p},\;0_{p\times (2p)}\right){\left(X^{T}\left(u_{i},v_{i}\right)W_{h}\left(u_{i},v_{i}\right)X\left(u_{i},v_{i}\right)\right)}^{-1}X^{T}\left(u_{i},v_{i}\right)\\ \; & \times W_{h}\left(u_{i},v_{i}\right)\left(y-\rho \mathrm{Wy}\right),\;i=1,2,\cdots ,n, \end{array}$$

where the subscript “L” represents the local-linear GWR estimation,

(15) $$\begin{array}{cc} \; & X(u_{i},v{}_{i})=\\ \; & \left(\begin{array}{ccccccccc} x_{11} & \cdots & x_{1p} & x_{11}\left(u_{1}-u_{i}\right) & \cdots & x_{1p}\left(u_{1}-u_{i}\right) & x_{11}\left(v_{1}-v_{i}\right) & \cdots & x_{1p}\left(v_{1}-v_{i}\right)\\ x_{21} & \cdots & x_{2p} & x_{21}\left(u_{2}-u_{i}\right) & \cdots & x_{2p}\left(u_{2}-u_{i}\right) & x_{21}\left(v_{2}-v_{i}\right) & \cdots & x_{2p}\left(v_{2}-v_{i}\right)\\ \vdots & \vdots & \vdots & \vdots & \vdots & \vdots & \vdots & \vdots & \vdots \\ x_{n1} & \cdots & x_{np} & x_{n1}\left(u_{n}-u_{i}\right) & \cdots & x_{np}\left(u_{n}-u_{i}\right) & x_{n1}\left(v_{n}-v_{i}\right) & \cdots & x_{np}\left(v_{n}-v_{i}\right) \end{array}\right), \end{array}$$

is the design matrix at $\left(u_{i},v_{i}\right)$, $0_{p\times (2p)}$ is the zero matrix of order p×(2p), and $W_{h}\left(u_{i},v_{i}\right)$ is the same diagonal calibration weights matrix in Equation (5). The resulting estimator of $M=\left(x_{1}^{T}\beta \left(u_{1},v_{1}\right),x_{2}^{T}\beta (u_{2},\right.{\left.v_{2}),\cdots ,x_{n}^{T}\beta \left(u_{n},v_{n}\right)\right)}^{T}$ is

$$\begin{array}{c} {\hat{M}}_{L}=S_{L}\left(h\right)\left(I_{n}-\rho W\right)y, \end{array}$$

where

(16) $$\begin{array}{cc} \; & S_{L}\left(h\right)=\\ \; & \left(\begin{array}{c} \left(x_{1}^{T},0_{1\times (2p)}\right){\left(X^{T}\left(u_{1},v_{1}\right)W_{h}\left(u_{1},v_{1}\right)X\left(u_{1},v_{1}\right)\right)}^{-1}X^{T}\left(u_{1},v_{1}\right)W_{h}\left(u_{1},v_{1}\right)\\ \left(x_{2}^{T},0_{1\times (2p)}\right){\left(X^{T}\left(u_{2},v_{2}\right)W_{h}\left(u_{2},v_{2}\right)X\left(u_{2},v_{2}\right)\right)}^{-1}X^{T}\left(u_{2},v_{2}\right)W_{h}\left(u_{2},v_{2}\right)\\ \vdots \\ \left(x_{n}^{T},0_{1\times (2p)}\right){\left(X^{T}\left(u_{n},v_{n}\right)W_{h}\left(u_{n},v_{n}\right)X\left(u_{n},v_{n}\right)\right)}^{-1}X^{T}\left(u_{n},v_{n}\right)W_{h}\left(u_{n},v_{n}\right) \end{array}\right). \end{array}$$

The same derivation for Equation (6) yields the following artificial spatial autoregressive model:

$$\begin{array}{c} \left(I_{n}-S_{L}\left(h\right)\right)y=\rho \left(I_{n}-S_{L}\left(h\right)\right)\mathrm{Wy}+\varepsilon . \end{array}$$

Substitute the instrumental estimator ${\hat{y}}_{w}$ of Wy in Equation (8) into the above model and obtain the local-linear GWR-based 2SLS (henceforth referred to as LGWR-2SLS) estimator of ρ as

(17) $${\hat{\rho }}_{L}={\left({\hat{y}}_{w}^{T}{\left(I_{n}-S_{L}\left(h\right)\right)}^{T}\left(I_{n}-S_{L}\left(h\right)\right){\hat{y}}_{w}\right)}^{-1}{\hat{y}}_{w}^{T}{\left(I_{n}-S_{L}\left(h\right)\right)}^{T}\left(I_{n}-S_{L}\left(h\right)\right)y.$$

The LGWR-2SLS estimators of the regression coefficient vectors are

(18) $$\begin{array}{cc} {\hat{\beta }}_{L}\left(u_{i},v_{i}\right)= & \left(I_{p},\;0_{p\times (2p)}\right){\left(X^{T}\left(u_{i},v_{i}\right)W_{h}\left(u_{i},v_{i}\right)X\left(u_{i},v_{i}\right)\right)}^{-1}\\ \; & \times X^{T}\left(u_{i},v_{i}\right)W_{h}\left(u_{i},v_{i}\right)\left(I_{n}-{\hat{\rho }}_{L}W\right)y,\;i=1,2,\cdots ,n. \end{array}$$

The fitted vector of the response variable at the sampling locations ${\left\{\left(u_{i},v_{i}\right)\right\}}_{i=1}^{n}$ can be expressed as

(19) $${\hat{y}}_{L}\left(h\right)={\left({\hat{y}}_{L1}\left(h\right),{\hat{y}}_{L2}\left(h\right),\cdots ,{\hat{y}}_{Ln}\left(h\right)\right)}^{T}=H_{L}\left(h\right)y,$$

where the hat matrix $H_{L}\left(h\right)$ is of the same form as that in Equation (13) except that $S_{G}\left(h\right)$ therein is replaced by $S_{L}\left(h\right)$, shown in Equation (16).

#### 2.2.3. Generating the Calibration Weights Matrix $W_{h}\left(u_{i},v_{i}\right)$ and Selecting the Bandwidth *h*

As is well known in the GWR literature [2,3], the elements in the calibration weights matrix in Equation (5) are generated by a kernel function K(t), which is usually taken to be the Gaussian kernel or the bisquare kernel, and the bandwidth *h* can be set to be fixed or adaptive. Specifically, given a focal sampling location $\left(u_{i},v_{i}\right)$, the weights with a fixed bandwidth at $\left(u_{i},v_{i}\right)$ are

(20) $$w_{jh}\left(u_{i},v_{i}\right)=K\left(\frac{d_{ij}}{h}\right),\;j=1,2,\cdots ,n,$$

where ${\left\{d_{ij}\right\}}_{j=1}^{n}$ are the distances (usually the Euclidean distance) from $\left(u_{i},v_{i}\right)$ to all of the sampling locations ${\left\{(u_{j},v_{j})\right\}}_{j=1}^{n}$. The weights with an adaptive bandwidth at $\left(u_{i},v_{i}\right)$ are commonly generated by the bisquare kernel, which shows

(21) $$w_{jk}\left(u_{i},v_{i}\right)=\left\{\begin{array}{cc} {\left(1-{\left(\frac{d_{ij}}{h_{ik}}\right)}^{2}\right)}^{2}, & d_{ij}\leq h_{ik},\\ 0, & d_{ij}>h_{ik}, \end{array}\right.\;\;\;j=1,2,\cdots ,n,$$

where the bandwidth $h_{ik}$ is the distance from $\left(u_{i},v_{i}\right)$ to its *k*-th nearest sampling location and is variable with $\left(u_{i},v_{i}\right)$. As shown by Gollini et al. [42], when the sampling locations ${\left\{(u_{i},v_{i})\right\}}_{i=1}^{n}$ are irregularly distributed over space, and the weights with an adaptive bandwidth perform better than those with a fixed bandwidth.

Throughout this paper, the AICc criterion [3] is employed to select the optimal bandwidth size in both GWR-2SLS and LGWR-2SLS estimation approaches. Specifically, let $H\left(h\right)$ be the hat matrix in either of the two estimation methods. The AICc score is computed by

(22) $$\mathrm{AICc}=\log\left(\frac{1}{n}y^{T}{\left(I_{n}-H\left(h\right)\right)}^{T}\left(I_{n}-H\left(h\right)\right)y\right)+\frac{n+\mathrm{tr}(H(h))}{n-2-\mathrm{tr}(H(h))},$$

where $H\left(h\right)=H_{G}\left(h\right)$ in Equation (13) for GWR-2SLS and $H\left(h\right)=H_{L}\left(h\right)$ in Equation (19) for LGWR-2SLS. The optimal bandwidth size, which we denote by $h_{0}$, is

(23) $$h_{0}=\arg\min_{h>0}\;\mathrm{AICc}\left(h\right).$$

**Remark 2**. *When the adaptive bandwidth is set, the optimal size of the parameter k, which is taken as a proxy of the adaptive bandwidth $h_{ik}$ at each $\left(u_{i},v_{i}\right)$, is selected by the AICc criterion. To avoid causing confusion, henceforth, we call k the bandwidth.*

### 2.3. Non-Iterative Multiscale Estimation Procedures for the SARGWR Model

#### 2.3.1. SARGWR-LL Procedure

Based on the LGWR-2SLS estimators ${\hat{\rho }}_{L}$ in Equation (17) and ${\hat{\beta }}_{L}\left(u_{i},v_{i}\right)$ in Equation (18) of the autoregressive coefficient ρ and the regression coefficients $\beta \left(u_{i},v_{i}\right)\;\left(i=1,2,\cdots ,n\right)$, the SARGWR-LL non-iterative multiscale estimation is formulated by the following steps:

(i) Let $h_{0L}$ be the optimal bandwidth size selected in the LGWR-2SLS estimation of the SARGWR model. Fix the instrumental estimator ${\hat{y}}_{w}$ of Wy in Equation (8) and ${\hat{\rho }}_{L}$ in Equation (17) at $h_{0L}$, which we denote by ${\hat{y}}_{w}\left(h_{0L}\right)$ and ${\hat{\rho }}_{L}\left(h_{0L}\right)$, respectively;

(ii) Let $\tilde{h}=c\;h_{0L}$ where c∈(0,1) is a constant called the bandwidth shrinking parameter. Compute the coefficient estimators ${\hat{\beta }}_{L}\left(u_{i},v_{i}\right)\;\left(i=1,2,\cdots ,n\right)$ in Equation (18) in which the bandwidth *h* is replaced by $\tilde{h}$ and ${\hat{\rho }}_{L}$ is substituted by ${\hat{\rho }}_{L}\left(h_{0L}\right)$. We denote the resulting estimators by

(24) $${\tilde{\beta }}_{\tilde{h}}\left(u_{i},v_{i}\right)={\left({\tilde{\beta }}_{1(\tilde{h})}\left(u_{i},v_{i}\right),{\tilde{\beta }}_{2(\tilde{h})}\left(u_{i},v_{i}\right),\cdots ,{\tilde{\beta }}_{p(\tilde{h})}\left(u_{i},v_{i}\right)\right)}^{T},\;i=1,2,\cdots ,n;$$

(iii) Fixing each $m\in \left\{1,2,\cdots ,p\right\}$ and substituting ${\hat{\rho }}_{L}\left(h_{0L}\right)$ and ${\left\{{\tilde{\beta }}_{j(\tilde{h})}\left(u_{i},v_{i}\right)\right\}}_{j=1,j\neq m}^{p}$ into the SARGWR model in Equation (1), we formulate the following artificial GWR model with a single spatially varying coefficient:

$$\begin{array}{cc} y_{i}^{*}\left(m\right) & \overset{\wedge }{=}y_{i}-{\hat{\rho }}_{L}\left(h_{0L}\right)\sum_{j=1}^{n}w_{ij}y_{j}-\sum_{j=1,j\neq m}^{p}{\tilde{\beta }}_{j(\tilde{h})}\left(u_{i},v_{i}\right)\;x_{ij}\\ \; & =\beta_{m}\left(u_{i},v_{i}\right)x_{im}+{\tilde{\varepsilon }}_{i},\;i=1,2,\cdots ,n. \end{array}$$

Calibrating the above model by the local-linear GWR estimation [38] with the optimal bandwidth size selected by the AICc criterion, we obtain the SARGWR-LL estimators of $\beta_{m}\left(u_{i},v_{i}\right)\;\left(i=1,2,\cdots ,n\right)$ as

$$\begin{array}{cc} {\hat{\beta }}_{m\left(h_{m}\right)}\left(u_{i},v_{i}\right)= & \left(1,0,0\right){\left(X_{m}^{T}\left(u_{i},v_{i}\right)W_{h_{m}}\left(u_{i},v_{i}\right)X_{m}\left(u_{i},v_{i}\right)\right)}^{-1}\\ \; & \times X_{m}^{T}\left(u_{i},v_{i}\right)W_{h_{m}}\left(u_{i},v_{i}\right)y^{*}\left(m\right),\;i=1,2,\cdots ,n, \end{array}$$

where

$$\begin{array}{c} X_{m}\left(u_{i},v_{i}\right)=\left(\begin{array}{ccc} x_{1m} & x_{1m}\left(u_{1}-u_{i}\right) & x_{1m}\left(v_{1}-v_{i}\right)\\ x_{2m} & x_{2m}\left(u_{2}-u_{i}\right) & x_{2m}\left(v_{2}-v_{i}\right)\\ \vdots & \vdots & \vdots \\ x_{nm} & x_{nm}\left(u_{n}-u_{i}\right) & x_{nm}\left(v_{n}-v_{i}\right) \end{array}\right),\;y^{*}\left(m\right)=\left(\begin{array}{c} y_{1}^{*}\left(m\right)\\ y_{2}^{*}\left(m\right)\\ \vdots \\ y_{n}^{*}\left(m\right) \end{array}\right), \end{array}$$

and $h_{m}$ is the optimal bandwidth size selected by the AICc criterion in which y and the hat matrix H(h) in the AICc score in Equation (22) are respectively replaced by $y^{*}\left(m\right)$ and

$$\begin{array}{c} H_{m}\left(h\right)=\left(\begin{array}{c} \left(x_{1m},0,0\right)P\left(u_{1},v_{1}\right)\\ \left(x_{2m},0,0\right)P\left(u_{2},v_{2}\right)\\ \vdots \\ \left(x_{nm},0,0\right)P\left(u_{n},v_{n}\right) \end{array}\right) \end{array}$$

with

$$\begin{array}{c} P\left(u_{i},v_{i}\right)={\left(X_{m}^{T}\left(u_{i},v_{i}\right)W_{h}\left(u_{i},v_{i}\right)X_{m}\left(u_{i},v_{i}\right)\right)}^{-1}X_{m}^{T}\left(u_{i},v_{i}\right)W_{h}\left(u_{i},v_{i}\right),\;i=1,2,\cdots ,n; \end{array}$$

(iv) Repeating Step (iii) for each of m=1,2,⋯,p, we finally obtain the SARGWR-LL estimators ${\hat{\beta }}_{1(h_{1})}\left(u_{i},v_{i}\right)$, ${\hat{\beta }}_{2(h_{2})}\left(u_{i},v_{i}\right)$, ⋯, ${\hat{\beta }}_{p(h_{p})}\left(u_{i},v_{i}\right)$ at each of ${\left\{(u_{i},v_{i})\right\}}_{i=1}^{n}$ with $h_{1}$, $h_{2}$, ⋯, $h_{p}$ being the final optimal bandwidth sizes of the *p* coefficients $\beta_{1}\left(u,v\right),\beta_{2}\left(u,v\right),\cdots ,$$\beta_{p}\left(u,v\right)$ in the SARGWR model in Equation (1), respectively;

(v) For each i=1,2,⋯,n, substituting ${\left\{{\hat{\beta }}_{m(h_{m})}\left(u_{i},v_{i}\right)\right\}}_{m=1}^{p}$ into the SARGWR model in Equation (1) yields the following artificial spatial autoregressive model:

$$\begin{array}{c} {\tilde{y}}_{i}\overset{\wedge }{=}y_{i}-\sum_{m=1}^{p}{\hat{\beta }}_{m\left(h_{m}\right)}\left(u_{i},v_{i}\right)\;x_{im}=\rho \sum_{j=1}^{n}w_{ij}y_{j}+{\tilde{\varepsilon }}_{i},\;i=1,2,\cdots ,n, \end{array}$$

or

$$\begin{array}{c} \tilde{y}=\rho \mathrm{Wy}+\tilde{\varepsilon }, \end{array}$$

where $\tilde{y}={\left({\tilde{y}}_{1},{\tilde{y}}_{2},\cdots ,{\tilde{y}}_{n}\right)}^{T}$ and $\tilde{\varepsilon }={\left({\tilde{\varepsilon }}_{1},{\tilde{\varepsilon }}_{2},\cdots ,{\tilde{\varepsilon }}_{n}\right)}^{T}$. Replacing Wy in the above model with its instrumental estimator ${\hat{y}}_{w}\left(h_{0L}\right)$ and re-estimating ρ by the least-squares method, we obtain the final SARGWR-LL estimator of ρ as

(25) $$\hat{\rho }={\left({\hat{y}}_{w}^{T}\left(h_{0L}\right){\hat{y}}_{w}\left(h_{0L}\right)\right)}^{-1}{\hat{y}}_{w}^{T}\left(h_{0L}\right)\tilde{y}.$$

**Remark 3**. *In step (ii), a bandwidth shrinking parameter c is introduced to shrink the optimal bandwidth size $h_{0L}$ selected in the LGWR-2SLS estimation, and the shrunk bandwidth $\tilde{h}$ is used to obtain the initial estimators of the regression coefficients. As noted by Fan and Zhang [8], a smaller bandwidth size will reduce estimation biases but increase estimation variances of the regression coefficients. The less biased initial estimators in Equation (24) are helpful in increasing the accuracy of the final estimators of the regression coefficients, while the increased variances can be expected to be smoothed out in the following step (iii).*

#### 2.3.2. SARGWR-GL Procedure

The above SARGWR-LL procedure takes the LGWR-2SLS estimators of the regression coefficients to be the initial estimators. As shown in Equation (18), the LGWR-2SLS estimators of the regression coefficients relate to a more complicated design matrix $X(u_{i},v{}_{i})$, shown in Equation (15), which should be re-set at each of the sampling locations ${\left\{\left(u_{i},v_{i}\right)\right\}}_{i=1}^{n}$. Therefore, computing ${\hat{\rho }}_{L}$ in Equation (17) and ${\hat{\beta }}_{L}\left(u_{i},v_{i}\right)\;\left(i=1,2,\cdots ,n\right)$ in Equation (18) is more time-demanding than computing their GWR-2SLS estimators ${\hat{\rho }}_{G}$ in Equation (10) and ${\hat{\beta }}_{G}\left(u_{i},v_{i}\right)\;\left(i=1,2,\cdots ,n\right)$ in Equation (11). Furthermore, the initial estimators of ρ and $\beta \left(u_{i},v_{i}\right)\;\left(i=1,2,\cdots ,n\right)$ might have less effect on their final estimators because $\beta \left(u_{i},v_{i}\right)\;\left(i=1,2,\cdots ,n\right)$ will be re-estimated by the local-linear GWR procedure and ρ will be re-estimated by the 2SLS estimation method. With these considerations, we replace the initial LGWR-2SLS estimators of ρ and $\beta \left(u_{i},v_{i}\right)\;\left(i=1,2,\cdots ,n\right)$ with their respective GWR-2SLS estimators and propose a simplified scenario of SARGWR-LL, which we refer, as mentioned in the introduction, to SARGWR-GL. The main steps of the SARGWR-GL procedure are as follows:

(i) Let $h_{0G}$ be the optimal bandwidth size selected in the GWR-2SLS estimation of the SARGWR model. Fix the instrumental estimator ${\hat{y}}_{w}$ of Wy in Equation (8) and ${\hat{\rho }}_{G}$ in Equation (10) at $h_{0G}$, which we denote by ${\hat{y}}_{w}\left(h_{0G}\right)$ and ${\hat{\rho }}_{G}\left(h_{0G}\right)$, respectively.

(ii) Let $\tilde{h}=c\;h_{0G}$. The estimators ${\hat{\beta }}_{\tilde{h}}\left(u_{i},v_{i}\right)\;\left(i=1,2,\cdots ,n\right)$ in Equation (24) are computed from ${\hat{\beta }}_{G}\left(u_{i},v_{i}\right)\;\left(i=1,2,\cdots ,n\right)$ in Equation (11), where *h* and ${\hat{\rho }}_{G}$ are replaced by $\tilde{h}$ and ${\hat{\rho }}_{G}\left(h_{0G}\right)$, respectively.

(iii) The steps followed are totally the same as those in Section 2.3.1, except that ${\hat{y}}_{w}\left(h_{0L}\right)$ in Equation (25) is replaced by ${\hat{y}}_{w}\left(h_{0G}\right)$.

#### 2.3.3. SARGWR-BF Procedure

Moreover, for the purpose of comparison, we accordingly formulate a backfitting-based multiscale estimation procedure for the SARGWR model, in which the GWR-2SLS estimation method is used in each iteration. We refer to this procedure as SARGWR-BF and describe its detailed steps in Appendix A because this part is less related to the main theme of this paper.

## 3. Simulation Study and Real-Life Example

In this section, a simulation study is conducted to assess the performance of the proposed SARGWR-LL and SARGWR-GL multiscale estimation methods for the SARGWR model. In particular, the proposed non-iterative multiscale estimation methods and the iterative multiscale estimation method SARGWR-BF described in Appendix A are compared in both the accuracy of the coefficient estimators and the computation efficiency. Furthermore, a real-life example based on Dublin voter turnout data is given to show the applicability of the proposed multiscale estimation methods.

### 3.1. Simulation Study

#### 3.1.1. Design of the Experiment

**(i)** 
**Spatial layout**

**Figure 1:**
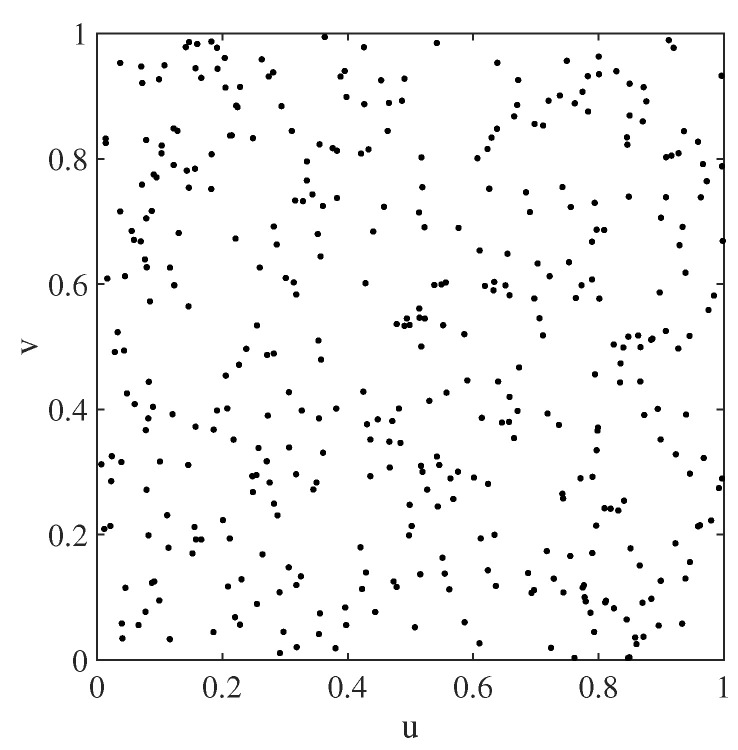
400 sampling points used in the simulation study.

We took the unit square [0,1] × [0,1] in a Cartesian coordinate system as the spatial region. Considering that the sampling locations in many practical problems are irregularly distributed over space, the sampling points ${\left\{\left(u_{i},v_{i}\right)\right\}}_{i=1}^{n}$ were designed in the way that each pair of $\left(u_{i},v_{i}\right)$ was independently drawn from the uniform distribution $U\left(0,1\right)$ with n=400. The sampling points used in the simulation are depicted in Figure 1, and they are fixed throughout the simulation.

**(ii)** 
**Model for generating the experimental data**

The following SARGWR model was considered:

(26) $$y_{i}=\rho \sum_{j=1}^{n}w_{ij}y_{i}+\beta_{1}\left(u_{i},v_{i}\right)+\beta_{2}\left(u_{i},v_{i}\right)x_{i2}+\beta_{3}\left(u_{i},v_{i}\right)x_{i3}+\varepsilon_{i},\;i=1,2,\cdots ,n,$$

where the row-standardized spatial weights matrix $W={\left(w_{ij}\right)}_{n\times n}$ was determined by the *l*-nearest neighbor procedure. As pointed out by Boots and Tiefelsdorf [43], numerous studies have found that irregular spatial tessellations share, on average, many topological properties with a hexagonal tessellation in which a given hexagon has, in general, six neighbors when we define that one hexagon is a neighbor of another hexagon if they have a common side. Accordingly, we took l=6 in the simulation study. Specifically, let $d_{ij}$ be the Euclidean distance between $\left(u_{i},v_{i}\right)$ and $\left(u_{j},v_{j}\right)$. Given each $\left(u_{i},v_{i}\right)$, for each of j=1,2,⋯,n with j≠i, if $d_{ij}\leq d_{il}$, we set ${\tilde{w}}_{ij}=1$; if $d_{ij}>d_{il}$, we set ${\tilde{w}}_{ij}=0$. Then the elements in W are defined by $w_{ij}={\tilde{w}}_{ij}/\sum_{j=1}^{n}{\tilde{w}}_{ij}$ for i,j=1,2,⋯,n with i≠j, and $w_{ii}=0\;\left(i=1,2,\cdots ,n\right)$ by convention. The observations ${\left\{x_{i2}\right\}}_{i=1}^{n}$ and ${\left\{x_{i3}\right\}}_{i=1}^{n}$ of the explanatory variables $X_{2}$ and $X_{3}$ were independently drawn from the uniform distribution $U(-\sqrt{3},\sqrt{3})$ and the standard normal distribution N(0,1), respectively. The model errors ${\left\{\varepsilon_{i}\right\}}_{i=1}^{n}$ were generated from the normal distribution $N(0,0.5^{2})$. We designated the three regression coefficients as

(27) $$\left\{\begin{array}{c} \begin{array}{cc} \; & \beta_{1}\left(u,v\right)=2\left(u+v\right);\\ \; & \beta_{2}\left(u,v\right)=\frac{4\sin\left(\sqrt{12{\left(u-0.5\right)}^{2}+12{\left(v-0.5\right)}^{2}}\right)}{\sqrt{12{\left(u-0.5\right)}^{2}+12{\left(v-0.5\right)}^{2}}};\\ \; & \beta_{3}\left(u,v\right)=64uv\left(1-u\right)\left(1-v\right). \end{array} \end{array}\right.$$

**Figure 2:**
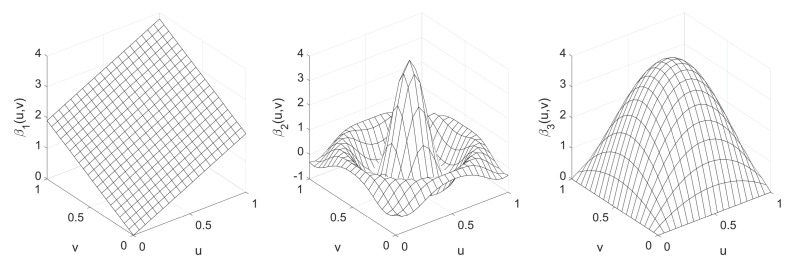
True surfaces of the three regression coefficients.

The true surfaces of the regression coefficients are shown in Figure 2, from which we can observe that their respective levels of spatial heterogeneity are obviously different. The autoregressive coefficient ρ was set to be from −0.9 to 0.9 with an increment of 0.3. When the values ${\left\{x_{i2},x_{i3}\right\}}_{i=1}^{n}$ of the explanatory variables $X_{2}$ and $X_{3}$ as well as ${\left\{\varepsilon_{i}\right\}}_{i=1}^{n}$ of the model errors have been drawn from their respective distributions, the observation vector y of the response variable is generated according to the matrix form of the model in Equation (2), i.e.,

$$\begin{array}{c} y={\left(I-\rho W\right)}^{-1}\left(M+\varepsilon \right), \end{array}$$

where $M={\left(x_{1}^{T}\beta \left(u_{1},v_{1}\right),x_{2}^{T}\beta \left(u_{2},v_{2}\right),\cdots ,x_{n}^{T}\beta \left(u_{n},v_{n}\right)\right)}^{T}$ with $x_{i}^{T}={\left(1,x_{i2},x_{i3}\right)}^{T}$, $\beta \left(u_{i},v_{i}\right)$$={\left(\beta_{1}\left(u_{i},v_{i}\right),\beta_{2}\left(u_{i},v_{i}\right),\beta_{3}\left(u_{i},v_{i}\right)\right)}^{T}$, and $\varepsilon ={\left(\varepsilon_{1},\varepsilon_{2},\cdots ,\varepsilon_{n}\right)}^{T}$.

**(iii)** 
**Designs of the other experimental items**

In both SARGWR-LL and SARGWR-GL, we set the shrinking parameter as c= 0.6, 0.7, 0.8, 0.9, and 1. The bisquare kernel with an adaptive bandwidth was used to generate the weights in Equation (21), and the optimal size of the bandwidth *k* was selected by the AICc criterion, where $\tilde{h}$ in step (ii) of both SARGWR-LL and SARGWR-GL was taken to be the integer part of $c\;h_{0L}$ or $c\;h_{0G}$.

**(iv)** 
**Indices for measuring accuracy of the coefficient estimators**

Each experimental setting was repeatedly run *N* times, where, in each replication, both model errors ${\left\{\varepsilon_{i}\right\}}_{i=1}^{n}$ and the observations ${\left\{x_{i2},x_{i3}\right\}}_{i=1}^{n}$ of the explanatory variables $X_{1}$ and $X_{2}$ were re-drawn from their respective distributions. Based on the coefficient estimators of the SARGWR model in Equation (26) in the *N* replications, we defined the following indices for measuring the accuracy of the coefficient estimators.

Given each of the SARGWR-LL, SARGWR-GL, and SARGWR-BF methods, let ${\hat{\rho }}_{\left(r\right)}$ be the estimator of the autoregressive coefficient ρ in the *r*-th replication. We take the mean of its estimators in the *N* replications,

$$\begin{array}{c} \mathrm{Mean}\left(\hat{\rho }\right)=\frac{1}{N}\sum_{r=1}^{N}{\hat{\rho }}_{\left(r\right)} \end{array}$$

as the final estimator of ρ and use the root mean square error (RMSE)

$$\begin{array}{c} \mathrm{RMSE}\left(\hat{\rho }\right)={\left(\frac{1}{N}\sum_{r=1}^{N}{\left({\hat{\rho }}_{\left(r\right)}-\rho \right)}^{2}\right)}^{\frac{1}{2}} \end{array}$$

to measure the estimation accuracy of ρ.

Similarly, let ${\hat{\beta }}_{j\left(r\right)}\left(u_{i},v_{i}\right)$ be the estimator of the *j*-th coefficient $\beta_{j}\left(u,v\right)$ at $\left(u_{i},v_{i}\right)$ in the *r*-th replication. The final estimator of $\beta_{j}\left(u_{i},v_{i}\right)$ is defined by

(28) $$\mathrm{Mean}\left({\hat{\beta }}_{j}\left(u_{i},v_{i}\right)\right)=\frac{1}{N}\sum_{r=1}^{N}{\hat{\beta }}_{j\left(r\right)}\left(u_{i},v_{i}\right),\;i=1,2,\cdots ,n.$$

In addition, the averaged root mean square errors (RMSEs) over the sampling points given by

$$\begin{array}{c} \mathrm{ARMSE}\left({\hat{\beta }}_{j}\left(u,v\right)\right)=\frac{1}{n}\sum_{i=1}^{n}{\left(\frac{1}{N}\sum_{r=1}^{N}{\left({\hat{\beta }}_{j\left(r\right)}\left(u_{i},v_{i}\right)-\beta_{j}\left(u_{i},v_{i}\right)\right)}^{2}\right)}^{\frac{1}{2}}, \end{array}$$

are used to measure the global estimation accuracy of $\beta_{j}\left(u,v\right)$.

In the simulation, we set N=200 to compute the above indices.

#### 3.1.2. Simulation Results with Analysis

**(i)** 
**Estimation accuracy of the autoregressive and regression coefficients**

With the experiment design in Section 3.1.1, the values of the estimation accuracy indices of the autoregressive and regression coefficients for SARGWR-LL and SARGWR-GL are reported in Table 1. For comparison, the related results from SARGWR-BF with the convergence threshold $\eta_{0}=0.01$ are also attached.

With regard to the autoregressive coefficient ρ, it is known from the fourth column of the table that its final estimators from the three multiscale estimation methods were comparable, and there was a trend where the estimators were somewhat smaller than their respective real values, especially when autocorrelation in the response variable is extremely high (i.e., the value of ρ is −0.9 and 0.9). In terms of the RMSE, the same trend is found for the estimation accuracy. However, for the small and moderate values of ρ, the three multiscale estimation methods all yield an accurate estimator of ρ. Moreover, the estimators of ρ yielded by the two non-iterative multiscale estimation models were rather robust to the variation of values of the shrinking parameter *c* in terms of both the mean and the RMSE indices.

For the regression coefficients $\beta_{j}\left(u,v\right)\;\left(j=1,2,3\right)$, SARGWR-LL generally yielded more accurate estimators than SARGWR-GL except for the case of ρ=0.9 for $\beta_{1}\left(u,v\right)$. The gain in the coefficient estimation accuracy for SARGWR-LL should have come from the initial estimators of regression coefficients where the local-linear GWR estimation procedure was used because, as shown by Wang et al. [38], the local-linear GWR procedure can yield more accurate estimators of the regression coefficients than the traditional GWR method especially when a regression coefficient is a linear function of spatial coordinates. However, there was no notable difference in the estimation accuracy between the two proposed non-iterative multiscale methods, even in the cases of ρ=−0.9 and 0.9, which demonstrates that the initial estimators of regression coefficients, as expected previously, do not produce notable effects on the accuracy of the final multiscale estimators of regression coefficients. Moreover, comparing the values of ARMSE for c=1 with those of *c* being less than 1, we know that, for both SARGWR-LL and SARGWR-GL, the improvement in accuracy of the final coefficient estimators can really be achieved by shrinking the optimal bandwidth size selected in the initial LGWR-2SLS or GWR-2SLS estimation in most cases, especially for the SARGWR-GL method. However, the values of ARMSE seemed robust for c= 0.6, 0.7, 0.8, and 0.9. This finding is useful in practice in that it provides a non-rigorous way for the analyst to choose the value of *c*. Among the three multiscale estimation methods, SARGWR-BF generally yielded the worst estimators of the regression coefficients in terms of RMSE, although the estimation accuracy could be improved by setting a smaller value of the convergence threshold $\eta_{0}$. The computation efficiency of the three multiscale estimation methods will be discussed further later.

**Figure 3:**
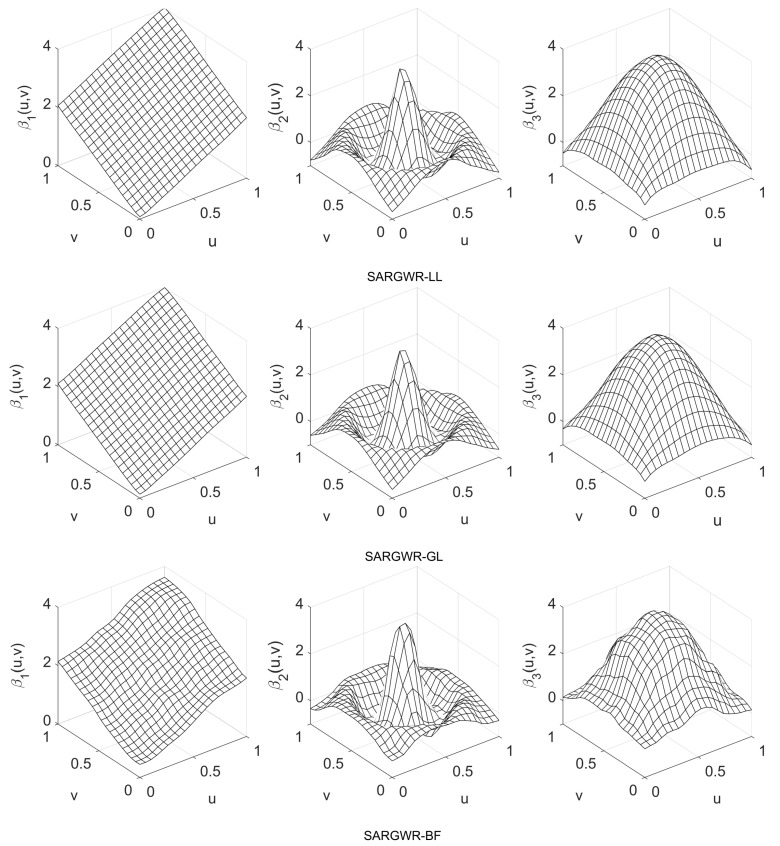
Estimated surfaces of the regression coefficients with c=0.7 and ρ=−0.9 by the three multiscale estimation methods.

**Figure 4:**
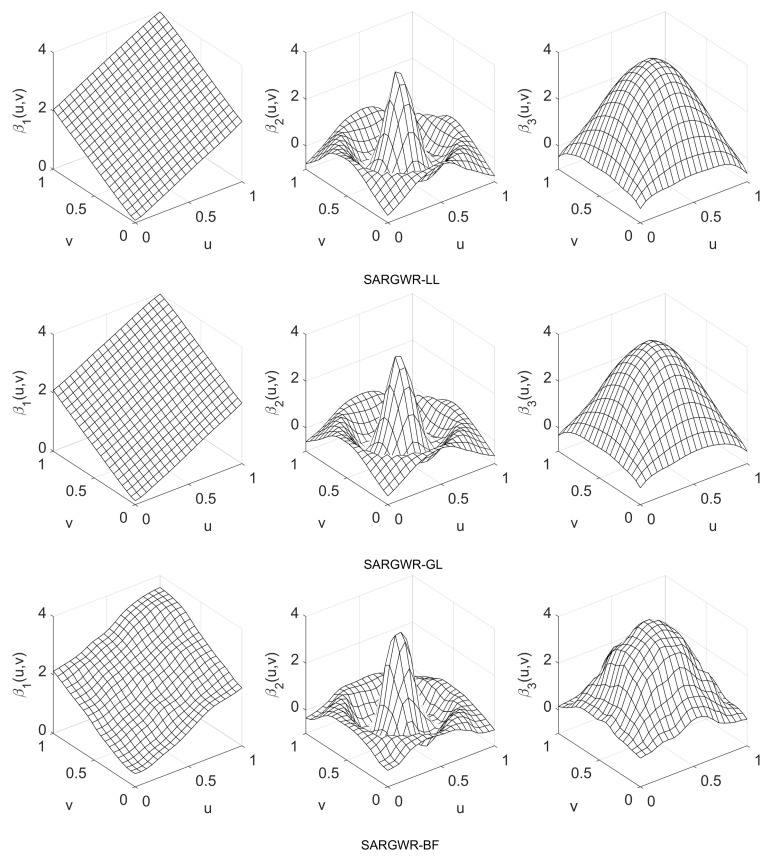
Estimated surfaces of the regression coefficients with c=0.7 and ρ=0 by the three multiscale estimation methods.

**Figure 5:**
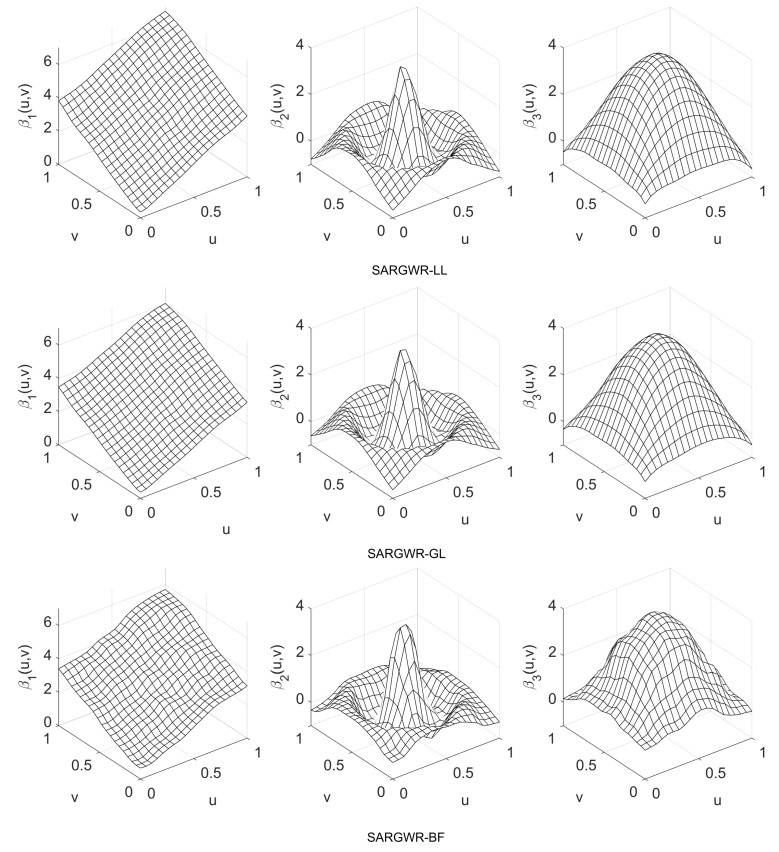
Estimated surfaces of the regression coefficients with c=0.7 and ρ=0.9 by the three multiscale estimation methods.

In order to visually show the performance of the three estimation methods in retrieving true surfaces of the regression coefficients, we depict in Figure 3, Figure 4 and Figure 5 the estimated surfaces of the regression coefficients via their respective final estimators defined in Equation (28) for the cases of ρ=−0.9,0,0.9 and c=0.7. The estimated surfaces in the other cases are all very similar, and we omitted them to save space. By comparing Figure 3, Figure 4 and Figure 5 with Figure 1, it can be observed that SARGWR-LL and SARGWR-GL retrieved the true surfaces of the regression coefficients more accurately than SARGWR-BF.

**(ii)** 
**Optimal bandwidth sizes of the regression coefficients**

**Figure 6:**
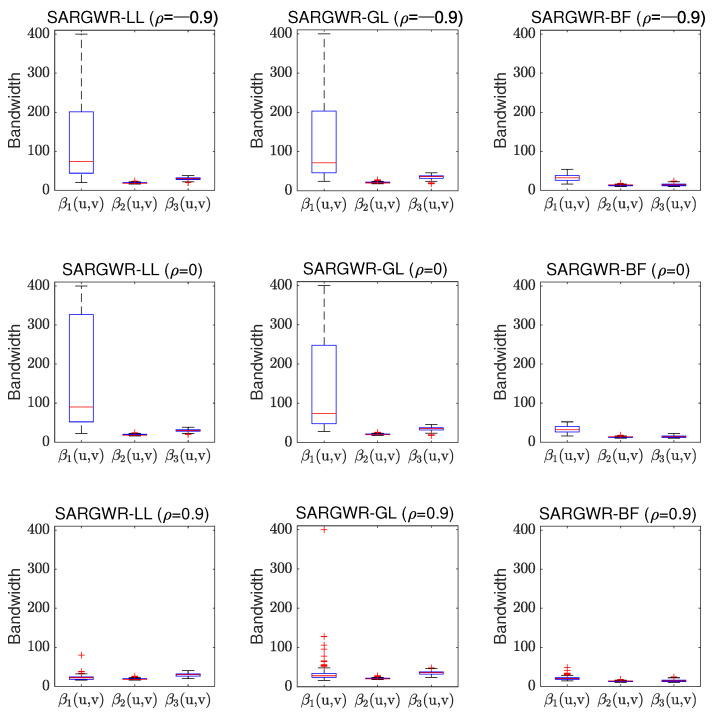
Boxplots of the optimal bandwidth sizes of the regression coefficients selected in the 200 experiment replications in the three multiscale estimation methods in the cases of c=0.7 and ρ=−0.9,0,0.9.

**Figure 7:**
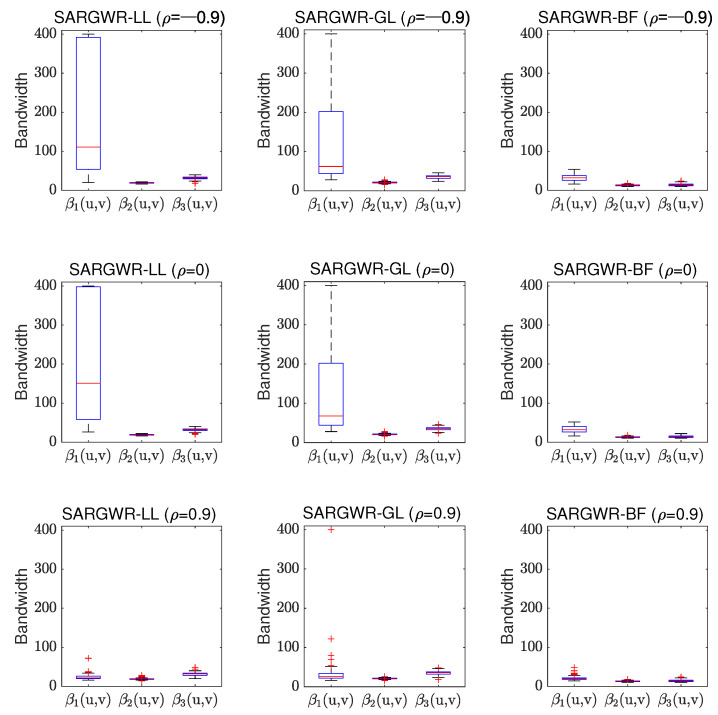
Boxplots of the optimal bandwidth sizes of the regression coefficients selected in the 200 experiment replications in the three multiscale estimation methods in the cases of c=1 and ρ=−0.9,0,0.9.

As mentioned in the introduction section, the optimal bandwidth size of each regression coefficient can characterize the underlying spatial scale of the corresponding explanatory variable, which was one of the main objectives for developing multiscale estimation methodologies for the GWR and the related models. Figure 6 and Figure 7 show shows the boxplots of the optimal bandwidth sizes of the three regression coefficients selected in the 200 experiment replications for SARGWR-LL, SARGWR-GL, and SARGWR-BF, respectively. Here, we only show the boxplots in the cases of c=0.7,1, and ρ=−0.9,0,0.9 to illustrate the impact of the shrinking parameter, *c*, and the autoregressive coefficient, ρ, on the optimal bandwidth sizes for the three multiscale estimation methods. The boxplots for the other experiment settings were all similar, which we omitted here.

It can be observed from Figure 6 and Figure 7 that the three multiscale estimation methods all yielded such variable-specific optimal bandwidth sizes that are consistent with the heterogeneity levels of the respective coefficients in almost all experiment settings. That is, the more heterogeneous the coefficient was, the smaller the optimal bandwidth size was, which demonstrates that the three methods can all correctly reveal the respective underlying spatial scales of the explanatory variables. Compared to the iterative estimation method SARGWR-BF, both non-iterative estimation methods SARGWR-LL and SARGWR-GL could better reflect the difference in heterogeneity among the three coefficients because the optimal bandwidth sizes from SARGWR-LL and SARGWR-GL show on the whole, an evident difference and the correct order of spatial heterogeneity levels among the three coefficients. However, the uncertainty of the optimal bandwidth sizes yielded by SARGWR-LL and SARGWR-GL for the least heterogeneous coefficient $\beta_{1}\left(u,v\right)$ was much larger than that produced by SARGWR-BF. The reason for causing this large uncertainty in SARGWR-LL and SARGWR-GL was perhaps due to the property that, theoretically, the optimal bandwidth size for a linear function in the local-linear kernel smoothing technique tends to infinity with the increase of the sample size [44]. For the other two non-linear coefficients, uncertainty in the optimal bandwidth size was comparable among the three multiscale estimation methods. Moreover, the figures show that the variable-specific optimal bandwidth sizes for the three methods were affected by the intensity of the spatial autocorrelation in the response variable, especially for the least heterogeneous coefficient $\beta_{1}\left(u,v\right)$. For example, when the very high positive spatial autocorrelation exists in the response variable (i.e., ρ=0.9), the medians of the optimal bandwidth sizes yielded by SARGWR-LL and SARGWR-GL for $\beta_{1}\left(u,v\right)$ become even slightly smaller than those for $\beta_{3}\left(u,v\right)$. However, less influence of the intensity of spatial autocorrelation on the optimal bandwidth sizes was observed for the SARGWR-BF method. In addition, comparing Figure 6 with Figure 7, we can see that the boxplots with c=0.7 are very similar to the corresponding boxplots with c=1 for both non-iterative multiscale estimation methods. This demonstrates that shrinking the optimal bandwidth size to obtain initial estimators of the regression coefficients had little impact on their respective final optimal bandwidth sizes.

**(iii)** 
**Computation efficiency**

As one of our main focuses for developing non-iterative multiscale estimation methods for the SARGWR model, computation efficiency is essential for SARGWR-LL and SARGWR-GL. According to the foregoing simulation, which was conducted by our writing the Matlab codes and carrying out the computation on our personal computer with AMD Ryzen 5 5600G @ 3.90GHz of CPU and 16GB of memory, the average time for running a replication was about 90 s for SARGWR-LL and about 80 s for SARGWR-GL. With the convergence threshold $\eta_{0}=0.01$, the SARGWR-BF method took about 210 s to run a replication, which is more than two times as much as the computing time that SARGWR-LL and SARGWR-GL took, respectively. In essence, both SARGWR-LL and SARGWR-GL are one-step SARGWR-BF by taking the initial estimators of the regression coefficients to be the LGWR-2SLS and the GWR-2SLS estimators with a shrunk optimal bandwidth size, respectively. Therefore, SARGWR-LL and SARGWR-GL should be more efficient than SARGWR-BF when the iteration times for implementing SARGWR-BF until convergence are greater than 1. Moreover, as expected, SARGWR-GL was more efficient than SARGWR-LL, and the difference in the computation time between SARGWR-LL and SARGWR-GL will become larger with the number of explanatory variables increasing. Furthermore, in view of the foregoing findings that both the estimation accuracy of the coefficients and the variable-specific optimal bandwidth sizes are all comparable between SARGWR-LL and SARGWR-GL, the SARGWR-BF provides a more efficient alternative for dealing with large data sets. Although computation time for an estimation method closely depends on the optimization of the codes written and the computation equipment used, the above comparison still makes sense in understanding the relative computation efficiency of the three multiscale estimation methods, in that the codes were written by ourselves and the computation was carried out on a same computer.

### 3.2. Real-Life Example

#### 3.2.1. Introduction to the Data Set with the Model Built

To demonstrate the applicability of the proposed non-iterative multiscale estimation methods, a Dublin voter turnout data set, which is publicly available in the R package attached in Gollini et al. [42], was analyzed in this section. This data set includes the observations of nine variables collected from 322 Electoral Divisions (EDs) of the Dublin area in the Irish 2004 Dáil elections, with the Cartesian coordinates (u,v) of each ED also provided. The nine variables are as follows:

- GenEl: percentage of the voting population in each ED;
- DiffAdd: percentage of the immigrant population one year ago in each ED;
- LARent: percentage of renters in each ED;
- SC1: percentage of people with high social class in each ED;
- Unempl: percentage of unemployed people in each ED;
- LowEduc: percentage of people without formal education in each ED;
- Age1: percentage of people aged from 18 to 24 in each ED;
- Age2: percentage of people aged from 25 to 44 in each ED;
- Age3: percentage of people aged from 45 to 64 in each ED.

What we are interested in is exploring spatial autocorrelation in the percentage of the voting population among the n=322 EDs and spatial heterogeneity of the impact of the eight variables on the percentage of voting population. For this purpose, we built the following SARGWR model:

$$\begin{array}{c} \begin{array}{c} {\mathrm{GenEl}}_{i}=\rho \sum_{j=1}^{n}{w_{ij}\mathrm{GenEl}}_{i}+\beta_{1}\left(u_{i},v_{i}\right)+\beta_{2}\left(u_{i},v_{i}\right)\mathrm{DiffAd}d_{i}+\beta_{3}\left(u_{i},v_{i}\right)\mathrm{LARen}t_{i}\\ \;\;\;\;+\beta_{4}\left(u_{i},v_{i}\right)\mathrm{SC}1_{i}+\beta_{5}\left(u_{i},v_{i}\right)\mathrm{Unemp}l_{i}+\beta_{6}\left(u_{i},v_{i}\right)\mathrm{LowEdu}c_{i}+\beta_{7}\left(u_{i},v_{i}\right)\mathrm{Age}1_{i}\\ \;\;\;\;+\beta_{8}\left(u_{i},v_{i}\right)\mathrm{Age}2_{i}+\beta_{9}\left(u_{i},v_{i}\right)\mathrm{Age}3_{i}+\varepsilon_{i},\;i=1,2,\cdots ,322, \end{array} \end{array}$$

where the spatial weights matrix $W={\left(w_{ij}\right)}_{n\times n}$ was formulated in the same way as that in the simulation study. That is, the elements in W were first determined by the binary way with the 6-nearest neighbor procedure used in the simulation study, where the Euclidean distance between the spatial coordinates of two EDs was used to determine the neighbors of each ED, and then were row-standardized. Moreover, all of the explanatory variables were standardized.

#### 3.2.2. Model Calibration with the Results

Both SARGWR-LL and SARGWR-GL methods were applied to the calibration of the above model, in which the bisquare kernel with an adaptive bandwidth was used to generate the calibration weights matrix in Equation (5), and the optimal bandwidth size was selected by the AICc criterion. Furthermore, the bandwidth shrinking parameter was set to c=0.7. For the purpose of comparison, the SARGWR-BF method was also used to calibrate the model, in which the convergence threshold was set to be $\eta_{0}=0.01$.

The estimated value of the autoregressive coefficient is 0.2547 for SARGWR-LL, 0.1810 for SARGWR-GL, and 0.1714 for SARGWR-BF, which are all positive and quantitatively similar, meaning that there exists positive spatial autocorrelation among the percentages of the voting population in the EDs. The running time on our personal computer is 56 s and 43 s for SARGWR-LL and SARGWR-GL, respectively, while SARGWR-BF took 68 s when the convergence threshold $\eta_{0}=0.01$ is reached.

The variable-specific optimal bandwidth sizes for the three multiscale estimation methods are listed in Table 2. In order to assess the impact of the shrinking parameter on the optimal bandwidth sizes of individual explanatory variables, the corresponding optimal bandwidth sizes selected in SARGWR-LL and SARGWR-GL with c=1 (i.e., the original optimal bandwidth size was not shrunk) are also reported in Table 2. It is known from the table that although the corresponding optimal bandwidth sizes among the three estimation methods are different from each other, their relative orders are roughly consistent, which provides information about the spatial scale of each explanatory variable. In particular, the influence of LARent, SC1, and LowEduc on the percentage of voting population (GenEl) is least heterogeneous because the three estimation methods all produce extremely large bandwidth sizes for these three explanatory variables, while the influence of DiffAdd is the most heterogeneous due to its very small bandwidth sizes yielded by the three estimation methods. Comparing the optimal bandwidth sizes with c=0.7 and the corresponding ones with c=1, we can observe that they are all comparable, which demonstrates, as shown in the simulation study, that the final optimal bandwidth sizes of the individual explanatory variables are very robust to the variation of the shrinking parameter for the SARGWR-LL and SARGWR-GL methods.

For the assessment of the goodness-of-fits and the ability to extract spatial autocorrelation of the three multiscale estimation methods, we computed the values of $R^{2}$ and Moran’s I of the residuals with the *p*-values derived by 200 randomly permuted residual samples. The values of $R^{2}$ are 0.7936 for SARGWR-LL, 0.8993 for SARGWR-GL, and 0.6896 for SARGWR-BF, respectively. The smaller value of $R^{2}$ for SARGWR-BF may be due to the relative inaccuracy of the regression coefficient estimators, as demonstrated in the simulation study. The values of Moran’s I of the residuals are 0.0654 with the *p*-value 0.020, 0.0555 with the *p*-value 0.055, and 0.0258 with the *p*-value 0.195 for SARGWR-LL, SARGWR-GL, and SARGWR-BF, respectively, showing that SARGWR-BF is of stronger ability for extracting spatial autocorrelation. In addition, for the comparison of goodness-of-fits of the three multiscale estimation methods with other possible models, we calibrated the corresponding SARGWR model with the GWR-2SLS method, GWR model with the traditional estimation method, and MGWR model, where the coefficients of the LARent, SC1 and LowEduc were assumed to be constant, with the two-step estimation method. The values of $R^{2}$ are 0.7060, 0.7135, and 0.7154 for SARGWR, GWR, and MGWR models, respectively, which are all smaller than the corresponding $R^{2}$ values of the multiscale estimation methods except for SARGWR-BF.

**Figure 8:**
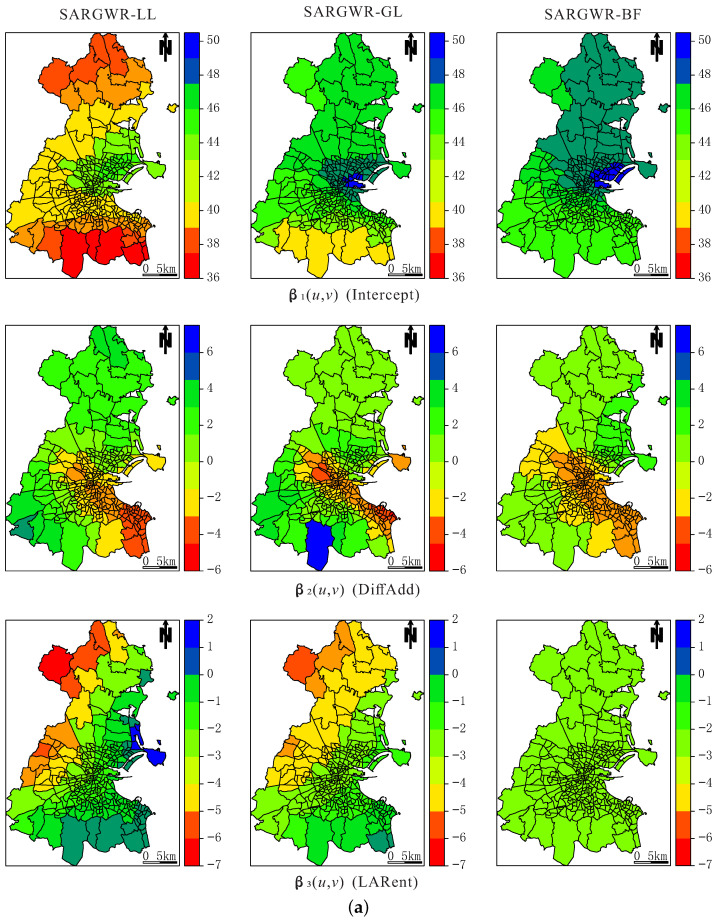

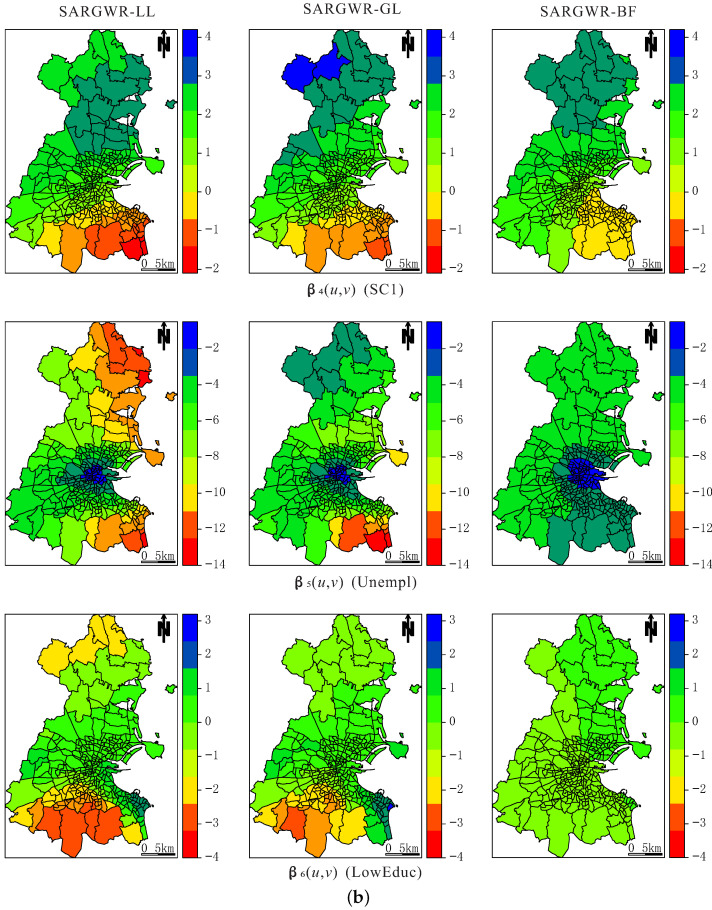

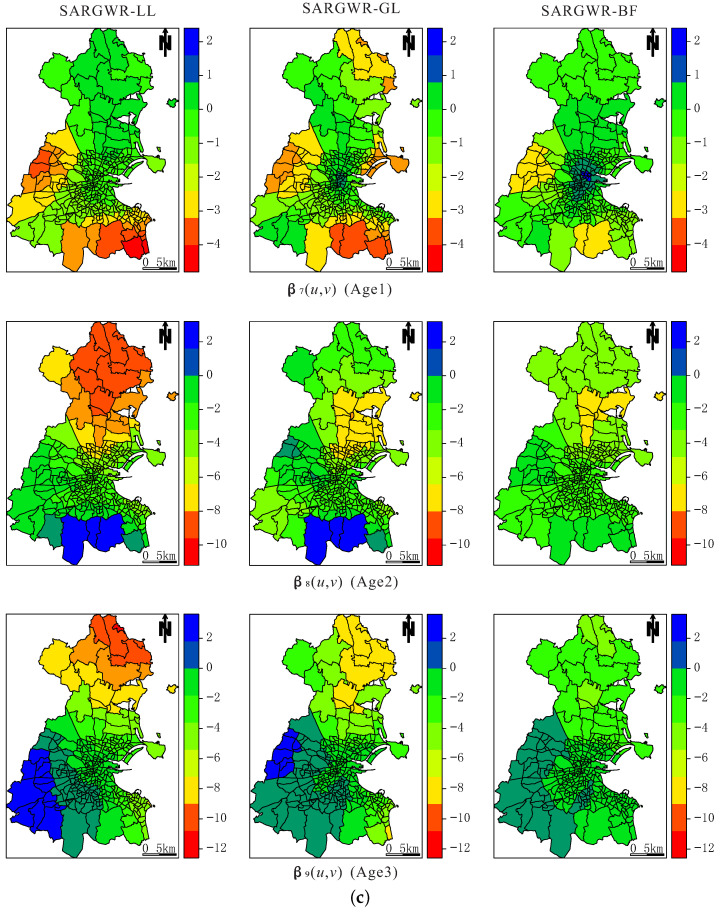
(**a**) Heatmaps of the estimators of the regression coefficients Intercept, DiffAdd, and LARent by the three multiscale estimation methods. (**b**) Heatmaps of the estimators of the regression coefficients SC1, Unempl, and LowEduc by the three multiscale estimation methods. (**c**) Heatmaps of the estimators of the regression coefficients Age1, Age2, and Age3 by the three multiscale estimation methods.

The estimators of the regression coefficients by the three estimation methods are shown via heat maps in Figure 8. It can be observed that the spatial patterns of individual coefficients are basically consistent among the three estimation methods, although some local differences exist, especially between the estimator of intercept by SARGWR-LL and those by SARGWR-GL and SARGWR-BF, which might be caused by the larger difference between the estimated values of spatial autoregressive coefficient ρ and the different multiscale estimation methods used. Relatively, however, the heat maps of the regression coefficient estimators produced by SARGWR-LL and SARGWR-GL are more similar.

Based on the heat maps of the regression coefficients, the impact of each explanatory variable on the response variable can be qualitatively interpreted. For example, the influence of Age3 on GenEl increases from north to south, showing a positive effect in the south area, especially in the southeast area, and a negative effect in the north area; Age1 has an evident negative impact on GenEl mainly in the south area, while the most positive influence of Age2 appears in this area; the influence of Unempl on GenEl is negative over the whole area with the least negative impact being on the center area. DiffAdd, whose coefficient is the most spatially heterogeneous, shows a weakly positive influence in the northern area but a strong negative influence in the center and southeast areas. Due to the very large optimal bandwidth sizes of the LARent, SC1, and LowEduc, their influence could be interpreted to be global. However, as will be discussed in the last section, a formal statistical test is still needed to verify that their corresponding coefficients are constant.

## 4. Conclusions and Discussion

Inspired by the cost-effective multiscale estimation approach proposed by Wu et al. [37] for calibrating GWR models and considering the importance of SARGWR models in the application, in this article, we proposed two non-iterative multiscale estimation methods called SARGWR-LL and SARGWR-GL, respectively, for SARGWR models based on their 2SLS estimation. The simulation study and real-life data analysis demonstrate that both SARGWR-LL and SARGWR-GL perform as well as or better than the iterative multiscale estimation method SARGWR-BF, not only in estimating the autoregressive coefficient and retrieving the underlying spatial patterns of the regression coefficients but also in revealing the spatial scales at which the explanatory variables operate. Most importantly, the proposed SARGWR-LL and SARGWR-GL methods were much more efficient than SARGWR-BF in terms of computational efficiency.

As aforementioned in Remark 1, the SARGWR model, which this paper has focused on, assumes a constant autoregressive coefficient. This assumption may be unrealistic for many real-life spatial data sets. Recently, Mei and Chen [41] have extended the SARGWR model to allow the autoregressive coefficient to vary over space and proposed a GWR-based 2SLS estimation method for the extended SARGWR model. It seems possible for both SARGWR-LL and SARGWR-GL multiscale estimation procedures to be extended to the SARGWR model with a spatially varying autoregressive coefficient, provided that the spatially varying autoregressive coefficient can be efficiently estimated by some local smoothing technique. This extension is worth investigating in view of the wide application background of the extended SARGWR model.

Moreover, SARGWR models assume that all of the regression coefficients vary over space, and their multiscale estimation can additionally provide information on the relative levels of spatial heterogeneity of the regression coefficients via variable-specific optimal bandwidth sizes. As pointed out by Yu et al. [45] and Fotheringham [46], formal statistical tests for identifying the constant coefficients and locally evaluating the significance of the influence of the explanatory variables at each spatial sampling point are also essential to regression-based local modeling. For the SARGWR model, Li et al. [47] proposed two kinds of tests to respectively identify whether spatial autocorrelation exists in the response variable and whether some of the regression coefficients are constant. Similar tests have also been proposed by Mei and Chen [41] for the extended SARGWR model. In these tests, there were too many null hypotheses for the regression coefficients to be considered, which makes it very complex to comprehensively identify the constant regression coefficients in the models. Taking into account the information of spatial heterogeneity of each regression coefficient provided by a multiscale estimation method as the prior information, i.e., an especially large bandwidth size of a regression coefficient means that this coefficient is possibly constant, we can formulate much fewer but more specific null hypotheses for testing the possible constant coefficients in a SARGWR or the extended SARGWR model. After the constant regression coefficients in a SARGWR model are well identified, a semi-parametric SARGWR model, where some regression coefficients are constant and others vary over space, is then formulated. It also seems possible to extend both SARGWR-LL and SARGWR-GL’s multiscale estimation methods to semi-parametric SARGWR models. These issues deserve to be studied in future research.

## Figures and Tables

**Table 1:** Values of the accuracy indices in the N=200 experiment replications.

			ρ		$\beta_{1}\left(u,v\right)$	$\beta_{2}\left(u,v\right)$	$\beta_{3}\left(u,v\right)$
ρ	Method	*c*	Mean	RMSE	ARMSE	ARMSE	ARMSE
−0.9	SARGWR-LL	0.6	−0.9455	0.0541	0.0885	0.1770	0.1429
		0.7	−0.9460	0.0543	0.0917	0.1725	0.1376
		0.8	−0.9462	0.0543	0.0933	0.1708	0.1363
		0.9	−0.9462	0.0542	0.0918	0.1707	0.1368
		1	−0.9459	0.0540	0.0880	0.1703	0.1375
	SARGWR-GL	0.6	−0.9564	0.0670	0.1134	0.2250	0.1710
		0.7	−0.9554	0.0654	0.1186	0.2071	0.1565
		0.8	−0.9555	0.0653	0.1216	0.1994	0.1499
		0.9	−0.9557	0.0654	0.1209	0.1995	0.1489
		1	−0.9563	0.0658	0.1210	0.2025	0.1489
	SARGWR-BF	NA	−0.9408	0.0532	0.1255	0.2075	0.2340
−0.6	SARGWR-LL	0.6	−0.6338	0.0449	0.0887	0.1767	0.1408
		0.7	−0.6343	0.0450	0.0918	0.1720	0.1355
		0.8	−0.6346	0.0451	0.0935	0.1704	0.1342
		0.9	−0.6345	0.0449	0.0915	0.1704	0.1346
		1	−0.6342	0.0448	0.0875	0.1699	0.1353
	SARGWR-GL	0.6	−0.6455	0.0590	0.1173	0.2245	0.1686
		0.7	−0.6449	0.0580	0.1222	0.2065	0.1542
		0.8	−0.6451	0.0580	0.1249	0.1989	0.1477
		0.9	−0.6453	0.0580	0.1247	0.1991	0.1467
		1	−0.6456	0.0581	0.1238	0.2020	0.1467
	SARGWR-BF	NA	−0.6315	0.0477	0.1280	0.2073	0.2335
−0.3	SARGWR-LL	0.6	−0.3231	0.0373	0.0895	0.1764	0.1392
		0.7	−0.3236	0.0375	0.0925	0.1718	0.1339
		0.8	−0.3240	0.0375	0.0944	0.1702	0.1327
		0.9	−0.3239	0.0374	0.0931	0.1701	0.1330
		1	−0.3236	0.0373	0.0887	0.1697	0.1338
	SARGWR-GL	0.6	−0.3341	0.0514	0.1223	0.2242	0.1670
		0.7	−0.3340	0.0508	0.1269	0.2061	0.1524
		0.8	−0.3343	0.0509	0.1296	0.1985	0.1458
		0.9	−0.3344	0.0508	0.1294	0.1987	0.1449
		1	−0.3345	0.0508	0.1286	0.2017	0.1448
	SARGWR-BF	NA	−0.3223	0.0435	0.1326	0.2071	0.2330
0	SARGWR-LL	0.6	−0.0135	0.0314	0.0939	0.1763	0.1381
		0.7	−0.0139	0.0315	0.0959	0.1716	0.1327
		0.8	−0.0142	0.0314	0.0983	0.1700	0.1315
		0.9	−0.0141	0.0315	0.0963	0.1699	0.1319
		1	−0.0139	0.0313	0.0927	0.1695	0.1327
	SARGWR-GL	0.6	−0.0223	0.0439	0.1299	0.2240	0.1656
		0.7	−0.0223	0.0435	0.1332	0.2058	0.1510
		0.8	−0.0226	0.0437	0.1362	0.1983	0.1444
		0.9	−0.0226	0.0435	0.1355	0.1985	0.1433
		1	−0.0227	0.0434	0.1350	0.2015	0.1434
	SARGWR-BF	NA	−0.0130	0.0392	0.1401	0.2069	0.2329
0.3	SARGWR-LL	0.6	0.2929	0.0274	0.1067	0.1762	0.1374
		0.7	0.2927	0.0274	0.1080	0.1715	0.1320
		0.8	0.2925	0.0274	0.1098	0.1699	0.1309
		0.9	0.2925	0.0273	0.1082	0.1699	0.1312
		1	0.2927	0.0273	0.1047	0.1694	0.1320
	SARGWR-GL	0.6	0.2885	0.0368	0.1436	0.2239	0.1646
		0.7	0.2884	0.0367	0.1470	0.2056	0.1499
		0.8	0.2881	0.0368	0.1498	0.1980	0.1432
		0.9	0.2882	0.0366	0.1487	0.1984	0.1422
		1	0.2881	0.0366	0.1481	0.2013	0.1423
	SARGWR-BF	NA	0.2952	0.0349	0.1546	0.2068	0.2327
0.6	SARGWR-LL	0.6	0.5860	0.0271	0.1632	0.1761	0.1372
		0.7	0.5858	0.0272	0.1650	0.1716	0.1320
		0.8	0.5857	0.0273	0.1663	0.1699	0.1307
		0.9	0.5858	0.0273	0.1652	0.1699	0.1311
		1	0.5858	0.0272	0.1629	0.1694	0.1317
	SARGWR-GL	0.6	0.5896	0.0306	0.1880	0.2237	0.1640
		0.7	0.5895	0.0307	0.1905	0.2057	0.1493
		0.8	0.5894	0.0308	0.1921	0.1980	0.1427
		0.9	0.5894	0.0307	0.1913	0.1983	0.1419
		1	0.5894	0.0307	0.1917	0.2011	0.1420
	SARGWR-BF	NA	0.5933	0.0296	0.1935	0.2073	0.2328
0.9	SARGWR-LL	0.6	0.8213	0.0835	1.7329	0.1835	0.1372
		0.7	0.8213	0.0835	1.7334	0.1777	0.1338
		0.8	0.8213	0.0835	1.7336	0.1770	0.1339
		0.9	0.8213	0.0835	1.7335	0.1776	0.1344
		1	0.8213	0.0835	1.7332	0.1773	0.1341
	SARGWR-GL	0.6	0.8376	0.0669	1.3931	0.2274	0.1640
		0.7	0.8375	0.0670	1.3938	0.2066	0.1494
		0.8	0.8374	0.0670	1.3949	0.1980	0.1441
		0.9	0.8374	0.0670	1.3951	0.1985	0.1425
		1	0.8374	0.0670	1.3947	0.2015	0.1418
	SARGWR-BF	NA	0.8374	0.0670	1.3951	0.2155	0.2388

Note: NA means “not applicable”.

**Table 2:** Variable-specific optimal bandwidth sizes yielded by the three multiscale estimation methods with the shrinking parameter c=0.7 and 1 for SARGWR-LL and SARGWR-GL.

		SARGWR-LL		SARGWR-GL		
Variable	Coefficient	c=0.7	c=1	c=0.7	c=1	SARGWR-BF
Intercept	$\beta_{1}\left(u,v\right)$	227	227	132	132	137
DiffAdd	$\beta_{2}\left(u,v\right)$	167	132	97	117	92
LARent	$\beta_{3}\left(u,v\right)$	297	287	322	322	322
SC1	$\beta_{4}\left(u,v\right)$	312	307	322	322	202
Unempl	$\beta_{5}\left(u,v\right)$	137	172	117	132	107
LowEduc	$\beta_{6}\left(u,v\right)$	247	247	247	247	322
Age1	$\beta_{4}\left(u,v\right)$	242	252	162	162	97
Age2	$\beta_{5}\left(u,v\right)$	167	192	117	127	107
Age3	$\beta_{6}\left(u,v\right)$	197	207	137	162	102

## Data Availability

The Dublin voter turnout data set is publicly available in the R package GWmodel via https://cran.r-project.org/web/packages/GWmodel/index.html (accessed on 22 June 2022).

## Appendix A. SARGWR-BF Iterative Multiscale Estimation of the SARGWR Model

In order to make a comparison of the estimation accuracy and especially the computation efficiency between the proposed SARGWR-LL and SARGWR-GL procedures and the backfitting-based multiscale estimation for the SARGWR model, we describe in detail the SARGWR-BF method in this appendix. The SARGWR-BF method here is, in fact, more efficient than the backfitting-based multiscale estimation for the SARGWR model in Chen et al. [17] because the GWR-2SLS estimation, rather than the profile maximum likelihood estimation in Chen et al. [17], is used to derive the estimators of the autoregressive coefficient and the regression coefficients in each iteration loop. Therefore, this kind of comparison can better highlight the advantage of the SARGWR-LL and SARGWR-GL methods in reducing the computation complexity if SARGWR-LL and SARGWR-GL are more efficient than SARGWR-BF. What follows are the main steps of the SARGWR-BF procedure.

(i) Set the initial value of the autoregressive coefficient ρ to be $\rho^{\left(0\right)}={\hat{\rho }}_{G}\left(h_{0G}\right)$ in Step (i) of SARGWR-GL and the initial estimators of the regression coefficients to be the GWR-2SLS estimators in Equation (11) with $h=h_{0G}$, that is,

$$\begin{array}{cc} {\hat{\beta }}^{\left(0\right)}\left(u_{i},v_{i}\right) & ={\left({\hat{\beta }}_{1}^{\left(0\right)}\left(u_{i},v_{i}\right),{\hat{\beta }}_{2}^{\left(0\right)}\left(u_{i},v_{i}\right),\cdots ,{\hat{\beta }}_{p}^{\left(0\right)}\left(u_{i},v_{i}\right)\right)}^{T}\\ \; & ={\left(X^{T}W_{h_{0G}}\left(u_{i},v_{i}\right)X\right)}^{-1}X^{T}W_{h_{0G}}\left(u_{i},v_{i}\right)\left(I_{n}-{\hat{\rho }}_{G}\left(h_{0G}\right)W\right)y,\;i=1,2,\cdots ,n. \end{array}$$

Fix the instrumental estimator of Wy as ${\hat{y}}_{w}\left(h_{0G}\right)$, which is described in Step (i) of SARGWR-GL.

(ii) Let ${\hat{\rho }}^{\left(t-1\right)}$ and ${\hat{\beta }}^{\left(t-1\right)}\left(u_{i},v_{i}\right)={\left({\hat{\beta }}_{1}^{\left(t-1\right)}\left(u_{i},v_{i}\right),{\hat{\beta }}_{2}^{\left(t-1\right)}\left(u_{i},v_{i}\right),\cdots ,{\hat{\beta }}_{p}^{\left(t-1\right)}\left(u_{i},v_{i}\right)\right)}^{T}\;(i=1,2,$⋯,n) be the *t*-th iteration values of ρ and $\hat{\beta }\left(u_{i},v_{i}\right)\left(i=1,2,\cdots ,n\right)$, respectively. Formulate for each of m=1,2,⋯,p the following univariate GWR model:

$$\begin{array}{cc} {\tilde{y}}_{i}^{\left(t\right)} & \overset{\wedge }{=}y_{i}-{\hat{\rho }}^{\left(t-1\right)}\sum_{j=1}^{n}w_{ij}y_{j}-\sum_{j=1}^{m-1}{\hat{\beta }}_{j}^{\left(t\right)}\left(u_{i},v_{i}\right)\;x_{ij}-\sum_{j=m+1}^{p}{\hat{\beta }}_{j}^{\left(t-1\right)}\left(u_{i},v_{i}\right)\;x_{ij}\\ \; & =\beta_{m}\left(u_{i},v_{i}\right)x_{im}+{\tilde{\varepsilon }}_{i},\;i=1,2,\cdots ,n. \end{array}$$

Calibrating the model by the GWR technique [2] and selecting the optimal bandwidth size by the AICc criterion, we obtain the updated estimators ${\hat{\beta }}_{m}^{\left(t\right)}\left(u_{i},v_{i}\right)$ of $\beta_{m}\left(u_{i},v_{i}\right)\;\left(i=1,2,\cdots ,n\right)$ and the optimal bandwidth size $h_{m}^{\left(t\right)}$ for each of m=1,2,⋯,p.

(iii) Let

$$\begin{array}{c} y_{i}^{*\left(t\right)}\overset{\wedge }{=}y_{i}-\sum_{j=1}^{p}{\hat{\beta }}^{\left(t\right)}\left(u_{i},v_{i}\right)\;x_{ij}=\rho \sum_{j=1}^{n}w_{ij}y_{j}+{\tilde{\varepsilon }}_{i},\;i=1,2,\cdots ,n, \end{array}$$

or

$$\begin{array}{c} y^{*\left(t\right)}={\left(y_{1}^{*\left(t\right)},y_{2}^{*\left(t\right)},\cdots ,y_{n}^{*\left(t\right)}\right)}^{T}=\rho \mathrm{Wy}+\tilde{\varepsilon }, \end{array}$$

where $\tilde{\varepsilon }={\left({\tilde{\varepsilon }}_{1},{\tilde{\varepsilon }}_{2},\cdots ,{\tilde{\varepsilon }}_{n}\right)}^{T}$. Replacing Wy in the above model by its instrumental estimator ${\hat{y}}_{w}\left(h_{0G}\right)$ and using the least-square procedure, we obtain the updated estimator of ρ as

$$\begin{array}{c} {\hat{\rho }}^{\left(t\right)}={\left({\hat{y}}_{w}^{T}\left(h_{0G}\right){\hat{y}}_{w}\left(h_{0G}\right)\right)}^{-1}{\hat{y}}_{w}^{T}\left(h_{0G}\right)y^{*\left(t\right)}. \end{array}$$

(iv) Iteratively carry out Steps (ii) and (iii) until a pre-specified convergence criterion is reached. Here, the following convergence criterion is considered. That is, define

$$\begin{array}{c} \eta \left(t\right)={\left(\frac{{∥{\hat{\rho }}^{\left(t\right)}\mathrm{Wy}-{\hat{\rho }}^{\left(t-1\right)}\mathrm{Wy}∥}^{2}+\sum_{j=1}^{p}{∥{\hat{f}}_{j}^{\left(t\right)}-{\hat{f}}_{j}^{\left(t-1\right)}∥}^{2}}{{∥{\hat{\rho }}^{\left(t\right)}\mathrm{Wy}∥}^{2}+\sum_{j=1}^{p}{∥{\hat{f}}_{j}^{\left(t\right)}∥}^{2}}\right)}^{\frac{1}{2}}, \end{array}$$

where

$$\begin{array}{c} {\hat{f}}_{j}^{\left(t\right)}={\left({\hat{\beta }}_{j}^{\left(t\right)}\left(u_{1},v_{1}\right)x_{1j},{\hat{\beta }}_{j}^{\left(t\right)}\left(u_{2},v_{2}\right)x_{2j},\cdots ,{\hat{\beta }}_{j}^{\left(t\right)}\left(u_{n},v_{n}\right)x_{nj}\right)}^{T},\;j=1,2,\cdots ,p. \end{array}$$

Given a threshold $\eta_{0}$, when $\eta \left(t\right)\leq \eta_{0}$ for some t≥1, we terminate the iteration process and take ${\hat{\rho }}^{\left(t\right)}$ as the final estimator of the autoregressive coefficient and ${\hat{\beta }}^{\left(t\right)}\left(u_{i},v_{i}\right)=({\hat{\beta }}_{1}^{\left(t\right)}\left(u_{i},v_{i}\right),{\hat{\beta }}_{2}^{\left(t\right)}\left(u_{i},v_{i}\right),\cdots ,$${\hat{\beta }}_{p}^{\left(t\right)}\left(u_{i},v_{i}\right){)}^{T}\left(i=1,2,\cdots ,n\right)$ as the final estimators of the regression coefficients with $h_{1}^{\left(t\right)},h_{2}^{\left(t\right)},\cdots ,h_{p}^{\left(t\right)}$ being their respective optimal bandwidth sizes.
